# High mobility group box 1 and its post-translational modifications: Molecular mechanisms underlying neurodegenerative disease pathogenesis

**DOI:** 10.4103/NRR.NRR-D-25-01091

**Published:** 2025-12-30

**Authors:** Jun Li, Naming Wu, Yifan Xiao, Yiyuan Xia

**Affiliations:** 1 Wuhan Pulmonary Hospital, Wuhan Institute for Tuberculosis Control, Wuhan, Hubei Province, China; 2 Department of Dermatology, Renmin Hospital of Wuhan University, Wuhan, Hubei Province, China; 3 Hubei Key Laboratory of Cognitive and Affective Disorders, Jianghan University, Wuhan, Hubei Province, China; 4 Department of Pathology and Pathophysiology, School of Medicine, Jianghan University, Wuhan, Hubei Province, China; 5 Institute of Biomedical Sciences, School of Medicine, Jianghan University, Wuhan, Hubei Province, China

**Keywords:** acetylation, Alzheimer’s disease, alpha-synuclein, blood–brain barrier, high mobility group box 1 protein, multiple sclerosis, neuroinflammatory diseases, Parkinson’s disease, RAGE, Toll-like receptors

## Abstract

High mobility group box 1 is a dynamic nuclear protein that acts as a damage-associated molecular pattern when released from cells and plays key roles in neurodegenerative diseases. This review comprehensively analyzes the related post-translational modifications that affect the dual functions of high mobility group box 1 in neuroinflammation and neuronal survival, including acetylation, phosphorylation, oxidation, S-nitrosylation, lactylation, and ubiquitination. Post-translational modifications play critical regulatory roles in high mobility group box 1 subcellular localization, release processes and the specificity of receptor binding. In Alzheimer’s disease, high mobility group box 1 exacerbates the disease through the Toll-like receptor 4/nuclear factor kappa B signaling pathway. Inhibition of high mobility group box 1 acetylation can alleviate neuroinflammation. Parkinson’s disease models indicate that the S-nitrosylation of Cys106 is essential for the secretion of high mobility group box 1, which contributes to dopaminergic degeneration through the activation of microglia. In multiple sclerosis, high mobility group box 1 obstructs remyelination by inhibiting the maturation of oligodendrocytes and activating pro-inflammatory pathways. In contrast, high mobility group box 1 can maintain autophagy and DNA repair functions, suggesting its protective role. Therapeutic strategies targeting high mobility group box 1 show potential benefits. Glycyrrhizic acid inhibits disulfide-linked high mobility group box 1, SIRT activators suppress acetylation, and anti-high mobility group box 1 antibodies neutralize extracellular isoforms, thereby improving the results of preclinical studies. However, the diverse functions of high mobility group box 1 and the lack of post-translational modification-specific biomarkers present challenges for clinical translation. Future research should aim to create selective inhibitors that can cross the blood-brain barrier to target harmful forms of high mobility group box 1, and establish post-translational modification-based biomarkers for early detection. This review emphasizes that accurately targeting of high mobility group box 1 post-translational modifications in neurodegenerative diseases could be a new approach that can interrupt neuroinflammatory cascades while maintaining neuroprotective functions.

**Facts**

• Post-translational modifications serve as molecular switches that regulate the transport of high-mobility group box 1 protein from the nucleus to the extracellular space in neurodegenerative diseases.

• Targeted therapies specific to pathogenic isoforms of high-mobility group box 1 protein demonstrate therapeutic potential without compromising physiological functions.

• Future treatment strategies need to achieve cell-type-specific delivery and establish biomarkers for the dynamic changes of post-translational modifications.

**Open questions**

• How do synergistic post-translational modifications coordinate and regulate the function of high-mobility group box 1 in specific types of neurons?

• Can brain-targeted delivery systems achieve selective inhibition of pathogenic isoforms of high-mobility group box 1 in human neurodegenerative diseases?

• What is the optimal therapeutic window for targeting post-translational modifications of high-mobility group box 1 at different stages of neurodegenerative pathologies?

## Introduction

Neurodegenerative diseases (NDDs) are defined by the gradual loss of neuronal structure and function, which in turn leads to irreversible neural decline and presents a major challenge to global health systems (Temple, 2023). The pathogenesis of NDDs links to the breakdown of neural repair mechanisms and impaired neural regeneration, ultimately resulting in the buildup of cellular damage and synaptic loss (Lin and Beal, 2006). In such circumstances, neurons in the central nervous system (CNS) undergo progressive deterioration (Claudino dos Santos et al., 2025; Torres-Rico et al., 2025), which gives rise to a spectrum of neurological deficits varying with the specific brain regions affected (Dileep et al., 2023; Kampmann, 2024). NDDs may be categorized based on molecular irregularities, anatomical localization, or clinical features, with examples including multiple sclerosis (MS), Alzheimer’s disease (AD), and Parkinson’s disease (PD) (Nikom and Zheng, 2023; Zhao et al., 2024). With the aging of the global population, especially in developed countries, the prevalence of NDDs is increasing (Culig et al., 2022). Globally, the prevalence of MS shows significant geographical differences (5–300 cases per 100,000 people), with a relatively high incidence rate at high latitudes (McGinley et al., 2021). The 2021 World Alzheimer’s Report emphasized that there are currently over 55.2 million people worldwide suffering from dementia, and this number is expected to reach 78 million by 2030 (Dubois et al., 2021). AD is the predominant type of dementia, accounting for 60%–80% of cases (Garre-Olmo, 2018). PD, the second most prevalent NDDs, has a significant increase in incidence rate after the age of 50 years, with an estimated 850,000 global patients according to the data of WHO in 2019 (Jiang et al., 2025). The challenges associated with other NDDs, including amyotrophic lateral sclerosis (ALS), Huntington’s disease (HD), and frontotemporal degeneration, are also increasing, causing considerable economic burdens to society (Jiang et al., 2025).

In clinical practice, the symptoms of these diseases are diverse. Individuals diagnosed with MS frequently report symptoms such as fatigue, blurred vision, optic neuritis (eye pain), limb weakness or sensory disturbances, dizziness, balance impairments, cognitive deficits, and bladder dysfunction, and the risk of depression and anxiety also increases (Marcus, 2022). AD is characterized by progressive cognitive decline, including memory loss and disorientation (Scheltens et al., 2021), whereas PD is characterized by abnormal movement such as resting tremors and bradykinesia (Tanner and Ostrem, 2024). Behavioral symptoms such as depression and hallucinations are common in many NDDs. Pathological changes related to proteins, such as tauinopathy (Zhang et al., 2024), α-synucleinopathies (Park et al., 2025), amyloidosis (Kaji et al., 2024), and the hyperphosphorylation of the TAR DNA-binding protein 43 (Babazadeh et al., 2023), typically occur several years before clinical symptoms and remain major challenges for the NDDs. In addition, the role of innate immune activation is still controversial. While the prevailing opinion suggests that neuroinflammation contributes to neurodegeneration, some research indicates that specific inflammatory signals might offer neuroprotective benefits (Yang et al., 2020; Chen et al., 2022). Although the precise cause of NDDs remains unclear, targeted treatment strategies for neuroinflammation may be a promising approach (Patani et al., 2023). In addition, synaptic degeneration, neuroinflammation, and oxidative stress collectively promote disease progression. The existence of overlapping pathological features between different NDDs suggests the possibility of common mechanisms and promotes research on early interventions, such as protein misfolding, neural regeneration therapy, and gene-based therapy (Jiang et al., 2025). A comprehensive understanding of these fields is crucial for advancing early diagnosis and developing effective treatment methods.

**Figure 1:**
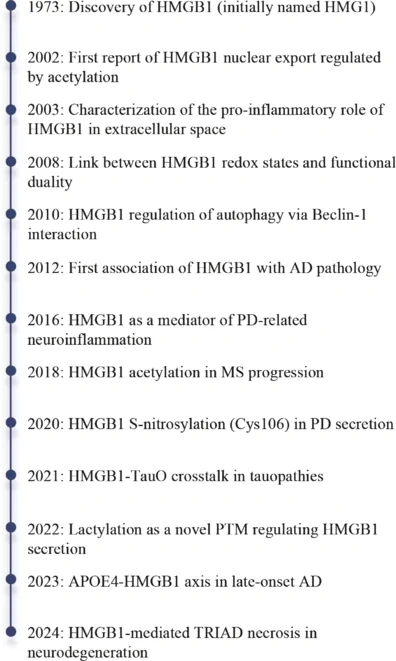
Timeline of HMGB1 research milestones in NDDs. This timeline highlights key milestones that connect the molecular properties of HMGB1 to its role in NDDs, including AD, PD, MS, and tauopathies. It underscores discoveries that have advanced both mechanistic understanding and therapeutic progress. The timeline illustrates a paradigm shift in HMGB1 research, moving from an initial focus on its nuclear DNA-binding function (1973–2002) to the recognition of its extracellular role as a damage-associated molecular pattern (2003–2008), and ultimately to the understanding of its PTM-dependent functional duality (2010–2024). This evolution supports the central argument of the review that the precise targeting of the PTMs of HMGB1 is crucial for disrupting the pathology of NDDs while preserving its neuroprotective functions. Later milestones (2020–2024) emphasize translatable findings, such as the APOE4-HMGB1 axis in AD and S-nitrosylation in PD—discoveries that provide actionable targets for biomarker development and drug design. AD: Alzheimer’s disease; APOE4: apolipoprotein E4; DAMP: damage-associated molecular pattern; HMGB1: high mobility group box 1; MS: multiple sclerosis; NDDs: neurodegenerative diseases; PD: Parkinson’s disease; PTM: post-translational modification.

High mobility group (HMG) proteins are nonhistone proteins that widely exist in the nucleus of eukaryotes (Hock et al., 2007). This protein family can be divided into three subclasses: HMGA, HMGB, and HMGN, which play important roles in regulating transcription, chromatin remodeling, and DNA replication (Hock et al., 2007). High mobility group box 1 (HMGB1), the most prevalent member of the HMGB family, has dual functions based on its subcellular localization. Extracellular HMGB1 is passively released or actively secreted from necrotic/stressed cells and plays a role as a damage-associated molecular pattern (DAMP). It binds to pattern recognition receptors and triggers significant inflammatory and immune responses. For example, the receptor for advanced glycation end products (RAGE), an important HMGB1 receptor, has been implicated in the pathogenesis of multiple NDDs, including AD, PD, and ALS (Kim et al., 2023; Baek et al., 2025). Extracellular HMGB1 is closely related to the pathogenesis of numerous CNS disorders, including epilepsy, AD, PD, ALS, MS, ischemic stroke, frontotemporal dementia (FTD), and traumatic brain injury. In recent years, an increasing number of studies have explored the treatment strategies for HMGB1 (such as glycyrrhizin, ethyl pyruvate, and neutralizing antibodies) (Kim et al., 2025b). While HMGB1 is a key driver of detrimental inflammation in these cases (Tan et al., 2024), emerging evidence also suggests that HMGB1 may play a beneficial role in promoting regeneration in some CNS diseases, emphasizing the complexity of its role in NDDs (**Figure 1**).

Post-translational modifications (PTMs) are fundamental mechanisms for regulating protein function, localization, and interaction, and they play a crucial role in the pathogenesis of diseases, including NDDs. Among PTM-sensitive proteins, HMGB1 exemplifies how PTMs act as molecular switches to determine functional outcomes (Lv et al., 2018). As a conserved nuclear DNA-binding protein and DAMP, HMGB1 has dual roles in modulating inflammation and immune responses, promoting or inhibiting innate immunity depending on its PTM status. For example, the functional activity, subcellular localization, extracellular release, and receptor binding of HMGB1 are regulated by PTMs such as acetylation, phosphorylation, methylation, and oxidation (Ge et al., 2024). These PTMs directly influence whether HMGB1 drives pro-inflammatory signaling, facilitates tissue repair, or performs other functions (Meng et al., 2024).

Although HMGB1 has been shown to be correlated with the pathogenesis of NDDs, there are still key gaps in knowledge concerning how specific PTMs accurately regulate the different roles of HMGB1 in different neurodegenerative environments. The current research on systematic comparisons across NDDs is limited, and there is no consensus on whether a specific PTM is disease-specific or stage dependent. Furthermore, existing therapeutic approaches targeting HMGB1 do not selectively explore its modification forms, which may help to explain its limited clinical efficacy and occasionally contradictory effects. Understanding these context-dependent PTM mechanisms, including their impact on HMGB1 structure, interactions, and inflammatory output, is crucial, as it has significant potential for identifying novel PTM-specific diagnostic biomarkers and developing therapeutic strategies. These strategies aim to selectively inhibit the harmful pro-inflammatory state of HMGB1 while retaining its beneficial function. In general, this review focuses on the new evidence of the mechanism of PTM-dependent regulation of the dual role of HMGB1 in NDDs, with a focus on its role in neuroinflammation, neuronal survival and regeneration. Unlike previous reviews that have broadly covered HMGB1 in CNS diseases, we focused on the mechanistic effects of PTMs on HMGB1 functions and signaling across major NDDs. Its unique contribution lies in the critical evaluation of HMGB1 as a tunable therapeutic target. We propose that PTM-specific interventions may enable the selective suppression of neurotoxic HMGB1 phenotypes while preserving their repair functions, which may promote the targeted treatment of NDDs in the future.

## Search Strategy

We conducted a comprehensive search of PubMed and Google Scholar from their inception to 2025. The search queries utilized Boolean operators (AND, OR) to combine keywords from three conceptual groups: (1) HMGB1 and its synonyms (e.g., “High Mobility Group Box 1 Protein,” “Amphoterin,” “HMG1 Protein”); (2) types of post-translational modifications (e.g., “Post-translational modification,” “PTM,” “Acetylation,” “Phosphorylation,” “Methylation,” “Oxidation”); and (3) neurodegenerative diseases (e.g., “Neurodegenerative disease,” “Alzheimer’s disease,” “Parkinson’s disease,” “Multiple sclerosis,” “Amyotrophic lateral sclerosis”). The search was limited to original research and review articles published in English. After screening, 84 documents were included for analysis, comprising both foundational and recent (2021–2025) highly cited papers.

## Structure, Functions, and Post-translational Modifications of High Mobility Group Box 1

### Molecular structure of high mobility group box 1

**Figure 2:**
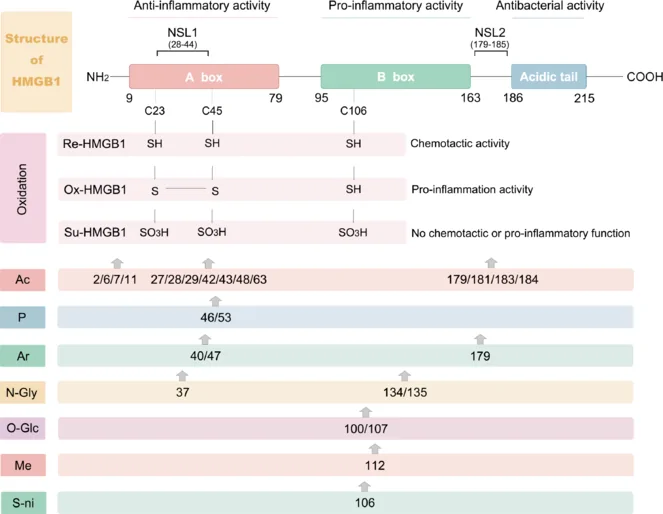
Structure and post-translational modification sites of HMGB1. HMGB1 is a 215-residue, approximately 30 kDa nuclear protein composed of two tandem DNA-binding motifs, known as the A-box and B-box, along with a highly acidic, negatively charged C-terminal tail. Three evolutionarily conserved cysteine residues (Cys23, Cys45, and Cys106) impart significant redox sensitivity to the molecule. The PTMs of HMGB1 include Ac, P, Ox, Me, Ar, N-Gly, and S-nitro. Ac: Acetylation; Ar: poly-ADP-ribosylation; HMGB1: high-mobility group box 1; Me: methylation; N-Gly: n-glycosylation; O-Glc: o-glcnacylation; Ox: oxidation; PTMs: post-translational modifications; P: phosphorylation; S-ni: S-nitrosylation.

The structure of HMGB1 is highly conserved across various species. Human HMGB1 is a 215-aa protein with A-box (9–79), B-box (95–163) and a C-terminal acidic tail (186–215). The A-box and B-box are capable of recognizing particular DNA structures and cisplatin-modified DNA duplexes (**Figure 2**). The extracellular B-box is capable of inducing pro-inflammatory activity, whereas the A-box antagonizes HMGB1 by binding to the B-box. The redox state of A-box modulates DNA binding. The steady location of HMGB1 in the nucleus is attributed to two nuclear localization signals (NLSs): NLS1 (28–44 amino acids) and NLS2 (179–185 amino acids). Moreover, the nuclear export signal is located in the DNA-binding domain.

Particular residues in the HMGB1 sequence are accountable for its interaction, binding, activity, and function. HMGB1 contains three cysteine residues (C23 and C45 in box A and C106 in box B). Changes in the redox environment can regulate the structure, location, and function of HMGB1. The two adjacent Cys23–Cys45 residues can rapidly form an intramolecular disulfide bond through oxidative folding, enhancing the stability of the folded full-length HMGB1 and altering the conformation of the A domain within HMGB1 (Park and Lippard, 2011). Mutations in Cys23 and Cys45 residues prevent HMGB1 from binding to the intracellular target protein Beclin 1, thereby inhibiting HMGB1 from initiating autophagy processes (Tang et al., 2010). A serine for cysteine substitution of C106 leads to HMGB1 translocation from the nucleus to the cytosol (Hoppe et al., 2006). The Cys106 residue in HMGB1 is crucial for its specific binding to Toll-like receptor 4 (TLR4), which triggers innate immune responses and cytokine release (Yang et al., 2010). Mutation of Cys106 completely blocks the formation of dimerized HMGB1. In the presence of excess ROS, HMGB1 dimerization mediated by Cys106 has a stronger DNA-binding capacity and can more effectively prevent DNA damage (Kwak et al., 2021). Shielding Cys106 via peptide-mediated approaches impairs the DNA-binding capacity of HMGB1 and significantly reduces nuclear p53 levels, thereby modulating p53 signaling in pulmonary arterial hypertension (Lawal et al., 2025).

The redox modifications of three cysteine residues in HMGB1 have a series of effects on its function. Reduced HMGB1 (Re-HMGB1), with all cysteine residues in thiol form, exhibits chemotactic activity. The formation of an intramolecular disulfide bond among Cys23, Cys45, and Cys106 yields “Ox-HMGB1.” Cytoplasmic Ox-HMGB1 promotes autophagy, whereas extracellular Ox-HMGB1 triggers inflammation. Mild oxidation which results in the formation of a C23–C45 disulfide bond while preserving a thiol group on C106 is essential for HMGB1 to induce phosphorylated NF-κB p65 and tumor necrosis factor-alpha (TNF-α) production (Yang et al., 2021b). In the hyperoxidized state, with all cysteines converted to sulfonic acid (Su-HMGB1), HMGB1 has no chemotactic or proinflammatory function (Kazama et al., 2008). The role of Cys106 in the function of HMGB1 has become increasingly clear in a series of studies (Kwak et al., 2021, 2023, 2025). Compared with monomeric HMGB1 protein, inflammatory stimuli such as lipopolysaccharide (LPS) and lipoteichoic acid promote HMGB1 self-association through reactive oxygen species (ROS)-mediated cysteine 106-cysteine 106 interactions. This self-association enhances nuclear factor kappa B (NF-κB) signaling and cytokine production by increasing the binding affinity of Toll-like receptor 2 (TLR2) and TLR4 and improving the effective delivery of pathogen-associated molecular patterns (PAMPs) (Kwak et al., 2025). Mutation of Cys106 in HMGB1 abolishes TNF-α-stimulating activity, despite the presence of Cys23–Cys45 disulfide bond (Kazama et al., 2008; Yang et al., 2010). In LPS-induced ALI inflammation, Pso covalently targets Cys106 of HMGB1, inhibits the HMGB1-TLR4 interaction, and suppresses cytokine storm generation (Wang et al., 2025). S-nitrosylation (SNO; the covalent binding of nitric oxide [NO] to cysteine thiols) modification at Cys106 by inducible nitric oxide synthase (iNOS)-derived NO is essential for HMGB1 secretion during inflammation. Deletion or inhibition of iNOS or the Cys106Ser mutation prevents LPS and/or poly(I:C)-induced secretion of HMGB1 (Yang et al., 2022b).

### Functions of high mobility group box 1

#### Intracellular functions of high mobility group box 1

Under physiological conditions, HMGB1 is predominantly located in the nucleus. As a DNA chaperone, HMGB1 binds to the minor groove of linear DNA, inducing helical bending that modulates DNA accessibility to cellular factors involved in DNA replication, DNA repair, chromatin remodeling, and transcription (Thwaites et al., 2019). By interacting with both DNA and histones, HMGB1 plays a crucial role in maintaining the structure of nucleosomes. It restricts the access of mutagens and photons to DNA, thereby preventing DNA damage. Additionally, HMGB1 can regulate gene expression by facilitating the sliding of nucleosome, a process that exposes DNA to the transcriptional machinery. HMGB1 binds to DNA and regulates chromatin structure (such as chromatin packaging and nucleosome stability), thereby providing binding sites for transcription factors and promoting gene accessibility (Chen et al., 2022a; Ren et al., 2023). For example, it can increase the accessibility of nucleosomes and transcription factor binding sites, which helps stabilize the transcription start sites (Kou et al., 2023). Furthermore, HMGB1 selectively promotes the transcription of genes involved in the regulation of transcription, osteoclast differentiation, and apoptotic processes (Liao et al., 2024). In the nucleus, HMGB1 can bind to or bend with the DNA or RNA with its transcriptional partners, control gene transcription and affect downstream targets, such as yes-associated protein, heat shock protein beta-1, and TNF-α. Functionally, the conditional depletion of HMGB1 in mouse cells or tissues results in genomic instability, telomere shortening, and the release of nucleosomes, which trigger inflammation and activate the innate immune response (Huang et al., 2014). An increase in circulating nucleosomes has been linked to immunopathological processes that can lead to lethal infections, autoimmune diseases, and tissue damage (Chen et al., 2012; Cavalier et al., 2021). These findings suggest the essential role of nuclear HMGB1 in maintaining nuclear homeostasis and controlling the release of nuclear DAMPs.

In mouse fibroblasts and human cancer cells, cytosolic HMGB1 plays a critical role in promoting autophagy in response to environmental stress (Tang et al., 2010; Livesey et al., 2012). Endogenous HMGB1 can promote autophagosome formation by competing with Bcl-2 for binding to Beclin 1, thereby directing Beclin 1 toward autophagosomes. This interaction and the subsequent maintenance of autophagy depend on the intramolecular disulfide bridge (C23/45) in HMGB1 (Kang et al., 2010; Tang et al., 2010). In murine models of inflammatory bowel disease, cytosolic HMGB1 forms complexes with BECN1 and ATG5 to protect proteins from calpain mediated pro-apoptotic cleavage (Zhu et al., 2015). In addition, cytosolic HMGB1-dependent autophagy plays a positive role in host defense against intestinal epithelial and myeloid cell infections (Tang et al., 2023).

HMGB1 is typically not localized to the membrane, except in specific contexts such as neurite outgrowth and platelet activation. During infection, oxidative stress induces HMGB1 translocation to the platelet membrane, where it binds neutrophil TLR4 or AGER to promote neutrophil extracellular traps (NETs) formation, exacerbating inflammatory damage in the brain, lung, and heart (Zhan et al., 2022; Zhang et al., 2022). HMGB1 derived from platelets also maintains neutrophil autophagy and NET release in systemic sclerosis. It further drives thrombosis through its interaction with TLR4 on platelets. Thus, membrane-associated HMGB1 may regulate intercellular communication in response to inflammatory stimuli.

#### Extracellular functions of high mobility group box 1 as a cytokine

HMGB1 can be released into the extracellular space through two mechanisms: active secretion and passive release. The active secretion of HMGB1 can be induced in multiple cell types, such as immune cells, fibroblasts, and epithelial or endothelial cells, by a range of stimuli, including exogenous microbial products, pathogen infections, and endogenous host signals. The passive release of HMGB1 occurs during cell death, differing from apoptosis. HMGB1 passively released by necrotic cells can activate the immune response and stimulate the production of inflammatory factors in cells (Li et al., 2018). Extracellular HMGB1, as a double-edged sword of innate immunity, initiates basic defense through its DAMP properties, but its sustained release or dysregulation can lead to chronic inflammation, tissue damage, and disease progression. Its functional diversity stems from the complexity of subcellular localization, oxidative state, and microenvironment interactions (Vladimirova et al., 2025).

The released HMGB1 can bind to a variety of receptors, including RAGE, TLR-2, TLR-4, TLR-9, and the type I interleukin (IL)-1 receptor. Subsequently, the NF-κB signaling pathway will be activated, promoting the release of pro-inflammatory cytokines such as TNF-α and IL-6. Extracellular HMGB1 amplifies inflammation in concert with other molecules such as DNA, RNA, receptors, or other DAMPs/PAMPs. For example, HMGB1 released during apoptosis forms complexes with nucleosomes. The complexes can interact with TLR2 in macrophages and dendritic cells (Urbonaviciute et al., 2008). The nucleosome complexes containing HMGB1 participate in the pathogenesis of systemic lupus erythematosus by enhancing the immunogenicity of nucleosomes released by apoptotic cells (Urbonaviciute et al., 2008). The complexes formed between HMGB1 and DNA increased cytokine production in macrophages, dendritic cells, and B cells by activating AGER and TLR9 (Ivanov et al., 2007). In addition, HMGB1-LPS not only enhances the production of TLR4-dependent cytokines, but also promotes the uptake of LPS through AGER (Deng et al., 2018). However, highly purified HMGB1 protein did not induce the production of inflammatory cytokines in mouse macrophage lines or in mixed rat glial cultures.

### Post-translational modifications of high mobility group box 1

#### Types of post-translational modifications affecting high mobility group box 1

PTMs of HMGB1, such as acetylation, phosphorylation, oxidation, methylation, poly-ADP-ribosylation, glycosylation, SNO, ubiquitination, and lactylation, affect its function and stability (**Additional Table 1**).

**Additional Table 1:** Types of PTMs of HMGB1 and their functional impacts

PTM type	Modified residue/region	Functional effect	Reference
Acetylation	Lys2, 6, 7, 11, 12, 27, 29; NLS1 (28-44), NLS2 (179-185)	Promotes nuclear export/extracellular release. Enhances TNF-α production.	Wei et al., 2023; Ge et al., 2024 Bielawski et al., 2024
		Inhibits DC maturation (↓ MHC-II, integrin αX).	Chen et al., 2018
		Pain, neuro-inflammation.	Dave et al., 2009; Gao et al., 2022; Wang et al., 2024b; Tsubota et al., 2025
		Sepsis, ALD, I/R injury.	Yang et al., 2022a, b; Xue et al., 2023
Phosphorylation	Ser46, Ser53	Decreases DNA-binding affinity.	Wang et al., 2024a
Oxidation	Cys23, Cys45, Cys106	Re-HMGB1: chemoattractant.	Tang et al., 2010
		Ox-HMGB 1: autophagy induction/extracellular inflammation.	Tang et al., 2010; Yang et al., 2010; Lee et al., 2014
		Su-HMGB1: loss of activity.	Kazama et al., 2008
Methylation	Lys112	Reduces DNA binding and leads to passive diffusion	Liao et al., 2024
N-glycosylation	Asn37, Asn134, Asn135	Reduces the binding to DNA and enhances release.	Kim et al., 2023
O-GlcNAcylation	Ser100, Ser107	Impairs DNA repair capacity.	Balana et al., 2021
Poly (ADP-ribosyl) ation	Glu40, Glu47, Glu179	Suppresses transcription.	Li et al., 2020
		Inhibits efferocytosis, sustaining inflammation.	Davis et al., 2012
S-nitrosylation	Cys106	Enhances secretion and pro-inflammatory effects.	Yang et al., 2022b
Ubiquitination	K48/K63 linkages	Targets for proteasomal degradation.	Sun et al., 2022; Du et al., 2024
		USP12/13/15 stabilize HMGB1 and promote autophagy or secretion.	Li et al., 2022; Shin et al., 2022

This table systematically compiles nine types of PTMs of HMGB1, detailing the specific amino acid residues or regions modified, the corresponding functional effects, and supporting references. The research indicates that different PTMs of HMGB1 can regulate its subcellular localization (e.g., nuclear export), binding ability with DNA, secretion levels, and involvement in inflammatory responses, autophagy, DNA repair, and other biological processes. In terms of application significance, this information provides a theoretical foundation for the targeted regulation of HMGB1 function in diseases such as inflammation-related disorders (e.g., sepsis, neuroinflammation), liver diseases (e.g., alcoholic liver disease), and ischemia/reperfusion injury. By intervening in specific PTMs of HMGB1, it may be possible to develop new therapeutic strategies for these related diseases. ALD: Alcoholic liver disease; DC: dendritic cell; HMGB1: high mobility group box 1 protein; I/R: ischemia/reperfusion; MHC-II: major histocompatibility complex class II; NLS: nuclear localization signal; PTMs: post-translational modification; TNF-α: tumor necrosis factor-α.

#### Impact of post-translational modifications on subcellular localization and activity of high mobility group box 1

The localization and activity of HMGB1 are regulated by a variety of PTMs. Acetylation, which predominantly occurs in the N-terminal region, enhances the ability of HMGB1 to bend DNA and prevents HMGB1 from re-entering the nucleus (Ge et al., 2024). HMGB1 shuttles between the nucleus and cytoplasm via acetylation and deacetylation of NLS1 and NLS2, which are mediated by histone acetyltransferase family proteins and histone deacetylase family proteins. Acetylation of lysine in the NLS can prevent the nuclear reentry of HMGB1 (Tang et al., 2016). A proteomic study has revealed that in addition to the NLS, significant acetylations occur in the N-terminal regions (Lys2, 6, 7, 11 and 27) of extracellular HMGB1 (Wei et al., 2023). Moreover, HMGB1 can bind to exportin 1 (XPO1) and be acetylated under oxidative stress, facilitating its translocation to the cytoplasm. In sepsis, lactate promotes HMGB1 lactylation and acetylation, leading to its translocation from the nucleus to the cytoplasm and subsequent release into circulation via exosome secretion (Yang et al., 2022a).

Methylation, glycosylation, phosphorylation, oxidation, and poly (ADP-ribosyl)ation also govern HMGB1 translocation and release in response to diverse stressors. Methylation of Lys112 in nuclear HMGB1 triggers a conformational change. This shift lowers the DNA-binding affinity of protein, enabling HMGB1 to diffuse passively out of the nucleus. Notably, overexpression of HMGB1 has been shown to affect the methylation of 28S rRNA (Liao et al., 2024). N-glycosylation of three specific HMGB1 residues—N37, N134, and N135—reduces the binding of protein to DNA and promotes its release. Additionally, O-GlcNAcylation modifies HMGB1 at S100 and S107, which impairs its ability to repair DNA (Balana et al., 2021).

Phosphorylation by protein kinase C (PKC) can target the HMGB1 A-box domain, directly influencing its DNA binding properties (Wang et al., 2024b). It is worth noting that nuclear magnetic resonance data confirm that PKC specifically phosphorylates the HMGB1 A-box at S46 and S53. This directly reduces its DNA binding affinity (Wang et al., 2024b). Oxidative modifications, which depend on the redox states of three key cysteine residues (Cys23, Cys45, and Cys106), determine the ability of HMGB1 to induce cytokine production or support tissue repair. In macrophages, the secretion of HMGB1 increases when the protein undergoes poly (ADP-ribosyl)ation (Davis et al., 2012; Yang et al., 2015a). Glutamate residues at positions 40, 47, and 179 have been identified as potential sites for this modification (Li et al., 2020). However, the regulatory mechanisms for managing these modifications under different conditions still need to be clarified.

#### Functional consequences of different post-translational modifications on high mobility group box 1

Acetylated HMGB1 has been shown to activate the production of pro-inflammatory cytokines (Bielawski et al., 2024). The substitution of six lysine residues with glutamines to mimic acetylated HMGB1 (HMGB1-M) revealed that HMGB1-M enhances TNF-α production in RAW 264.7 macrophages while reducing integrin αX and MHC-II expression on DCs, suggesting that it promotes inflammation and partially inhibits DC maturation (Chen et al., 2018). These findings are further supported by evidence that various oxidative stresses under pathological conditions can induce the release of hyperacetylated HMGB1, as confirmed in animal models (Dave et al., 2009). Upon toluene diisocyanate exposure, acetylation at lysine 12 and 29 of HMGB1 is increased, triggering the secretion of HMGB1. TLB inhibited Aβ_25–35_-induced acetylation of lysine 12 on HMGB1 through activating SIRT3/SOD2 signaling pathway, thereby restoring redox homeostasis and suppressing neuroinflammation in AD (Gao et al., 2022). Once released, acetylated HMGB1 acts extracellularly to trigger pain signals, linking them to nociceptive pathways (Tsubota et al., 2025). In the context of sepsis, lactate has been identified as a key driver of HMGB1 lactylation and acetylation in macrophages, which is mediated through p300/CBP, Hippo/YAP, SIRT1, and GPR81 pathways, ultimately increasing endothelial permeability via exosomal HMGB1 release and highlighting lactate signaling as a potential therapeutic target (Yang et al., 2022a). In alcohol-associated liver disease (AALD), oxidized HMGB1 derived from hepatocytes promotes inflammation through RAGE signaling in myeloid cells, whereas acetylated HMGB1 appears to counteract these harmful effects (Ge et al., 2024). Acetylation of HMGB1 amplifies the severity of oxygen-glucose deprivation/reperfusion injury by facilitating its translocation and release, resulting in elevated levels of pro-inflammatory cytokines, intensified oxidative stress, and increased hepatocellular apoptosis (Xue et al., 2023). Multiple signaling pathways are involved in the acetylation and deacetylation of HMGB1, including the interferon (IFN)-Janus kinase (JAK)-signal transducer and activator of transcription 1 (STAT1) pathway and the SIRT1 pathway.

The redox state of HMGB1 also plays a critical role in its function. Re-HMGB1 functions as a chemoattractant, while oxidation introduces a Cys23–Cys45 disulfide bond, forming Ox-HMGB1, which promotes intracellular autophagy and extracellular inflammation. In contrast, hyperoxidized HMGB1 (Su-HMGB1), with all cysteines converted to sulfonic acids, loses both chemotactic and pro-inflammatory functions (Kazama et al., 2008).

Further expanding the regulatory landscape of HMGB1, other PTMs also modulate its activity. Poly-(ADP)-ribosylated HMGB1 suppresses gene transcription and markedly inhibits efferocytosis in macrophages, thereby promoting inflammation (Davis et al., 2012). SNO has been shown to enhance HMGB1 secretion and proinflammatory effects (Yang et al., 2022b). RNF186 functions as an E3 ubiquitin ligase that targets cytoplasmic HMGB1 for ubiquitination with both lysine 48 (K48) and K63 linkages, thereby promoting its proteasomal degradation (Du et al., 2024). Ubiquitination generally promotes HMGB1 degradation and promotes disease progression (Sun et al., 2022). However, ubiquitin-specific protease (USP)-12 can deubiquitinate and stabilize HMGB1, promoting autophagy through its interaction with HMGB1 (Li et al., 2022). USP13 stabilizes HMGB1 against ubiquitin-mediated degradation, and its overexpression promotes HMGB1 nucleocytoplasmic translocation and secretion—effects blocked by the selective USP13 inhibitor Spautin-1 (Shin et al., 2022). Lactylated and acetylated HMGB1 is actively released from macrophages within exosomes, subsequently increasing endothelial permeability (Yang et al., 2022a). In sepsis-associated acute kidney injury, lactate-induced HMGB1 lactylation in macrophages promotes NET formation via the cGAS/STING pathway (Wei et al., 2025).

HMGB1 PTMs have distinct cell-type specificities that significantly affect its biological activities. After being stimulated by LPS, HMGB1 in bone marrow-derived macrophages (BMDMs) is oxidized to form disulfide bonds via PrxI/II catalysis. Oxidized HMGB1 is secreted by CRM1-mediated unconventional pathway, and its oxidation level affects the inflammatory activity (Kwak et al., 2020). In foam cells, oxidized HMGB1 may further be involved in cholesterol metabolism through regulating lipophagy (Robichaud et al., 2021). In contrast, in colorectal cancer (CRC) cells, HMGB1 PTMs switch to acetylation at K29 mediated by the cytoplasmic acetyltransferase P300 together with disulfide bonding, which are further upregulated by MSI2 that can enhance both HMGB1 translation and acetylation, leading to HMGB1 release and modulating dendritic cell maturation and antitumor immunity (Meng et al., 2024). The differences in HMGB1 PTMs between BMDMs/foam cells and CRC cells are due to the existence of cell-specific regulators (e.g., PrxI/II/CRM1 in macrophages versus MSI2/P300 in CRC) and cell-specific functional requirements (e.g., inflammatory and metabolic regulation in macrophages versus immune evasion in cancer) (Kwak et al., 2020). Therefore, the cellular context determines the pattern of HMGB1 modifications and downstream functional outputs. Being aware of this specificity is important for understanding the regulation of HMGB1 in pathophysiological processes.

Although substantial progress has been made in the field of HMGB1 PTMs, there are still several key gaps that need to be filled. First, most studies have focused on a single PTM (Kou et al., 2023; Wang et al., 2024c), but the crosstalk between concurrent modifications and their impact on HMGB1 function remain unclear. Second, although animal models have achieved some valuable results, additional clinical studies are needed to confirm these observations (Dong et al., 2022; Liu et al., 2023). Future research can prioritize the following areas. First, efforts should focus on elucidating the interactions among different PTMs using advanced proteomic and structural biology approaches. Second, the development of specific inhibitors or activators targeting key enzymes involved in HMGB1 modification is essential for precisely regulating their activity. Third, exploring HMGB1 PTMs as therapeutic targets for various inflammatory conditions, such as sepsis, NDDs, and cancer, is critical. Advancing research in this field will deepen our understanding of HMGB1 biology and lead to the discovery of new methods for clinical intervention.

## Role of High Mobility Group Box 1 in Neurodegenerative Diseases

### Multiple sclerosis

MS is a chronic autoimmune demyelinating disease defined by neuroinflammation in the CNS. Studies show that people with MS have significantly higher levels of HMGB1 in their peripheral blood than healthy individuals—and this elevation ties to increased pro-inflammatory cytokine levels and disease progression (Sternberg et al., 2016). In experimental autoimmune encephalomyelitis (EAE)—the principal animal model for MS—HMGB1 expression peaks during the onset phase in spinal cord tissue, serum, and cerebrospinal fluid (CSF) (Uzawa et al., 2013). Notably, as EAE advances, HMGB1 translocates from the nucleus to the cytoplasm in CNS-resident cells (e.g., neurons, microglia, and astrocytes), signaling its active release during inflammatory activation (Kim et al., 2025a).

HMGB1 contributes to the onset and progression of MS/EAE through multiple pathways. It promotes neuroinflammation by activating microglia and astrocytes, which in turn triggers the release of pro-inflammatory cytokines. This sequence of events establishes a self-sustaining inflammatory loop that exacerbates neuronal damage. HMGB1 released from oligodendrocyte precursor cells significantly impairs BBB integrity. This facilitates the infiltration of the CNS by CD4^+^ T cells. Using oligodendrocyte progenitor cell-specific HMGB1 deletion models, these findings were corroborated. Reduced immune cell infiltration, diminished demyelination, and milder EAE symptoms were exhibited by these models (Kim et al., 2025a). Furthermore, extracellular HMGB1 impedes the maturation of oligodendrocyte precursor cells into myelin-producing oligodendrocytes by activating NF-κB through TLR2 signaling. It directly obstructs remyelination efforts (Rouillard et al., 2022).

The HMGB1/TLR4/NF-κB signaling axis plays a crucial role in the pathogenesis of MS. HMGB1 binds to TLR4 and subsequently activates the MyD88/TRAF6 cascade, which triggers NF-κB phosphorylation and the upregulation of pro-inflammatory genes (Yuan et al., 2025). For example, matrine alleviates MS/EAE by targeting this pathway. It reduces the severity of the disease, inhibits inflammatory reactions, reduces demyelination, and suppresses the production of pro-inflammatory cytokines. The key mechanism involves downregulating the expression of HMGB1, TLR4, MyD88, and TRAF6, blocking NF-κB p65 phosphorylation, and increasing IκB-α levels. These findings were validated through experiments based on macrophages (Chu et al., 2021). Similarly, liraglutide exerts neuroprotective effects in a cuprizone induced MS mouse model. It enhances myelin regeneration, improves behavioral outcomes, and reduces cellular damage. These benefits link to activating the AMPK/SIRT1 pathway, stimulating autophagic flux, inhibiting the NLRP3 inflammasome, and regulating the TLR4/NF-κB axis. The AMPK inhibitor dorsomorphin can partially reverse these effects, highlighting the therapeutic potential of liraglutide (Ammar et al., 2022). In addition to TLR4, HMGB1 also drives pyroptosis of microglia and other cells through the TLR4/NF-κB axis. In MOG35-55-treated models, the increase in HMGB1 secretion is consistent with the increase in pro-inflammatory cytokine release and cell damage. Inhibition of HMGB1 can alleviate these outcomes and enhance its value as a therapeutic target (Feng et al., 2025).

The release of HMGB1 by astrocytes is also involved in disease regulation. Interleukin-33 (IL-33) knockout leads to P300/CBP related factors binding to HMGB1 and an increase in HMGB1 acetylation in the astrocyte nuclei resulting in HMGB1 release. However, IL-33 could reverse the elevated level of HMGB1 that is caused by TNF-α. The relevant study has proposed that the IL-33 pathway of HMGB1 may be a new treatment approach for EAE (Xiao et al., 2025). Furthermore, in astrocytes, HMGB1 binds to RAGE and triggers the release of sonic hedgehog (shh) via the JNK/p38/STAT3 signaling pathway. It is worth noting that shh treatment can slow down the progression of EAE and reduce demyelination. This indicates that HMGB1 plays a dual role in EAE/MS. It drives inflammation while potentially supporting neuroprotection through shh (Xiao et al., 2021). Importantly, the dual function of HMGB1 is not limited to MS. During neural development, it promotes the growth of neural processes and cell migration. In diseases such as AD, PD, and MS, it drives neuroinflammation through RAGE and TLR. However, in HD, it may play a neuroprotective role. Understanding these pathological mechanisms is crucial for developing treatments for NDDs (Fang et al., 2012).

### Alzheimer’s disease

AD is a progressive and incurable NDD characterized by cognitive decline. More and more evidence identifies HMGB1 as a key mediator in AD. It connects the pathological features, neuroinflammation, and neuronal damage of AD. Research has shown that HMGB1 colocalizes with amyloid-beta (Aβ) plaques in the brains of AD patients. Its levels are significantly elevated in both CSF and serum (Seol et al., 2024). The transgenic AD model (TG app/PS1) showed a decrease in HMGB1 levels in the nuclei of frontal cortex neurons and microglia, while an increase in cytoplasmic HMGB1. This indicates that HMGB1 translocates from the nucleus to the cytoplasm.

This process occurs before extracellular secretion and is consistent with Aβ deposition and aging. The above results indicate a temporal link between dynamic HMGB1 changes and pathological progression of AD (Seol et al., 2024). In clinical practice, elevated levels of HMGB1 and soluble RAGE have been observed in the serum of AD patients and mild cognitive impairment (MCI) patients. These levels correlate with Aβ concentrations and blood–brain barrier (BBB) dysfunction markers, such as soluble thrombomodulin.

HMGB1 drives AD progression via multiple pathways. The most notable way is through participation in extracellular signal transduction and interaction with key receptors. Extracellular, HMGB1 binds to TLR4 and RAGE, triggering activation of NF-κB pathway. This activation enhances the activity of microglia, promotes the release of pro-inflammatory cytokines, and increases the accumulation of Aβ. Recent evidence further demonstrates that IL-6 drives cognitive decline in mice by triggering TLR4 dependent neuroinflammation and neurodegeneration. This points to an axis that may intersect with HMGB1 signaling (Shentu et al., 2024). Additionally, the HMGB1-TLR4 axis activates PKCα, which phosphorylates Ku70 at Ser77/78. This can impair DNA repair and trigger transcription inhibition-induced atypical death (TRIAD) (Tanaka et al., 2021). On the contrary, Tau oligomers (TauO) induce HMGB1 release from neurons and glial cells, promoting cellular aging via the p38-MAPK/NF-κB pathway. Glycyrrhetinic acid can inhibit HMGB1, reduce the number of senescent cells, and alleviate neuroinflammation. This indicates the interaction between Tau pathology and HMGB1 (Gaikwad et al., 2021).

APOE4 accelerates the development of AD, the most common form of dementia, by facilitating the translocation of HMGB1 through the cell membrane of hippocampal neurons and subsequently releasing it. The APOE4-HMGB1 axis contributes to the exacerbation of various AD-related pathological processes, such as increased Tau phosphorylation, glial cell proliferation, and neurodegeneration. Notably, the blockade of HMGB1 mitigates all of these conditions, reaffirming the pro-pathogenic role of HMGB1 in AD. According to the study by Koutsodendris et al. (2023), HMGB1 is identified as a downstream mediator of APOE4. Additionally, HMGB1 compromises BBB integrity and tight junction function by binding to occludin-1 and other small molecules. This disruption allows pro-inflammatory molecules and toxins to enter the brain, thereby accelerating AD pathology (Nishibori et al., 2020). Elevated serum HMGB1 levels have been correlated with Aβ levels and BBB dysfunction in patients with AD and MCI, linking vascular insult to neuroinflammation (Nishibori et al., 2020). Furthermore, thrombin, a procoagulant enzyme, exacerbates BBB breakdown in conjunction with HMGB1, further integrating vascular and inflammatory pathways in AD (Festoff et al., 2016).

The accumulation of intracellular Aβ can trigger primary TRIAD necrosis in neurons. During this process, the release of HMGB1 promotes the spread of secondary necrosis and the aggregation of amyloid plaques (Okazawa, 2021). TRIAD necrosis is facilitated by the loss of function of the TEAD-YAP protein. HMGB1 exacerbates this process and leads to a self-sustaining damage cycle (Homma et al., 2024).

On the basis of multimodal analysis, HMGB1 has been identified as an AD-related aging gene in astrocytes (Sha et al., 2025). Environmental factors also participate in regulating the activity of HMGB1. For example, excessive alcohol consumption by adolescents regulates the neuroimmune signaling pathway through HMGB1, leading to AD, such as changes, in adulthood. In preclinical models, blocking HMGB1 can reverse ethanol induced Tau protein hyperphosphorylation and cognitive impairment (Fisher et al., 2025). Additionally, air pollutants such as ozone disrupt HMGB1 activity through the pulmonary cerebral circulation, promoting the activation of astrocytes (Ahmed et al., 2024).

Strategies targeting HMGB1 and its related pathways hold great potential for treating AD pathology and improving cognitive function. Research has shown that anti-HMGB1 antibodies alleviate AD symptoms by reducing DNA damage, decreasing amyloid accumulation, and mitigating cognitive impairment (Tanaka et al., 2021). Glycyrrhizic acid has also been found to alleviate ethanol-induced AD-like pathology and slow down TauO-related aging (Fisher et al., 2025). The microRNA miR-216a-5p exerts protective effects by inhibiting the HMGB1/NF-κB signaling pathway, leading to enhanced learning and memory in a mouse model of AD (Shao, 2021). Another compound, trilobatin, functions by activating SIRT3/SOD2, preventing HMGB1 acetylation, restoring redox balance, and inhibiting the HMGB1/TLR4/NF-κB pathway (Gao et al., 2022). Furthermore, activation of the delta-opioid receptor suppresses the secretion and translocation of HMGB1. Overexpression of delta-opioid receptor reduces microglial inflammatory responses via the HMGB1-NF-κB pathway (Xu et al., 2025). Electroacupuncture enhances cognitive function in SAMP8 mice by inhibiting HMGB1/TLR4 and RAGE/NADPH signaling, thereby reducing fibrin/Aβ deposition and BBB permeability (Wang et al., 2023).

In summary, HMGB1 plays a central mediator role in AD. It connects Aβ/Tau pathology, neuroinflammation, BBB damage, and neuronal death. HMGB1 dysfunction is influenced by genetic factors and environmental conditions, making it a promising multi-target candidate for altering the progression of AD. In addition, biomarkers such as serum HMGB1 and soluble RAGE may be helpful for AD diagnosis and progression monitoring.

### Parkinson’s disease

PD is the most common neurodegenerative motor disorder, characterized by the abnormal aggregation of alpha-synuclein (α-syn) in dopaminergic neurons of the substantia nigra. HMGB1 plays a complex role in PD by regulating neuronal function and the immune response. Preclinical research has shown promising prospects for targeting HMGB1 (Paudel et al., 2020b; De-Giorgio et al., 2024). However, a significant challenge remains: the non-selective inhibition of all HMGB1 isoforms, which hinders the progress of clinical applications. Further exploration of subtype-specific mechanisms is urgently needed to advance the treatment of PD (Tian et al., 2023).

Elevated levels of HMGB1 are a mark of PD. Compared with healthy controls, the serum HMGB1 levels in PD patients were significantly increased, and positively correlated with disease duration, Hoehn-Yahr (HY) stage, Unified Parkinson’s Disease Rating Scale scores, and severity of cognitive impairment. On the contrary, they are negatively correlated with the thickness of the inner plexiform layer of macular ganglion cells and the Montreal Cognitive Assessment scores (Liang et al., 2024). Combining HMGB1 measurements with the thickness of inner plexiform layer can improve the diagnostic accuracy for cognitive impairment in PD (Liang et al., 2024). In addition, HMGB1 and TLR4 are upregulated in peripheral blood, especially in patients with advanced disease, long course of illness, or poor treatment response. At the same time, the levels of downstream mediators (MyD88, NF-κB, and TNF-α) also increased. This identifies the HMGB1-TLR4 axis as a key pathway and potential diagnostic biomarker for PD progression (Yang et al., 2018). Elevated levels of HMGB1 were also detected in CSF and post-mortem substantia nigra tissue (Santoro et al., 2016). There is a moderate correlation between serum HMGB1 levels and high-sensitivity C-reactive protein levels, supporting its use as a supplementary biomarker for PD diagnosis (Baran et al., 2019).

Research has confirmed that HMGB1 is a key molecular link between chronic neuroinflammation and dopaminergic neuron degeneration in PD. When HMGB1 is released into the extracellular space, it binds to RAGE and TLR4 in microglia and astrocytes, activating downstream pro-inflammatory signaling pathways (Razali et al., 2023). This process continuously induces the production of pro-inflammatory cytokines (TNF-α, IL-1β, IL-6, and IL-17) and ROS, thereby creating a neurotoxic microenvironment. Ultimately, this leads to the loss of dopaminergic neurons in the substantia nigra pars compacta and impairs the integrity of BBB (Sasaki et al., 2016).

HMGB1 is involved in regulating the activation of microglia. Its inhibition (e.g., through A-box protein) suppresses excessive activation of microglia by restoring the immunosuppressive CD200-CD200R pathway and reducing T cell infiltration and pro-inflammatory Th17 differentiation (Tian et al., 2020). SNO of HMGB1 at Cys106 by iNOS-derived NO is essential for its active secretion, enhancing its pro-inflammatory and neurodegenerative effects (Liang et al., 2024). Cytoplasmic translocation can be regulated by the deacetylation of SIRT2 at lysine 43, promoting the release of HMGB1 and neuroinflammation (Xing et al., 2024). Moreover, the TRIAD (a necrotic subtype) driven by HMGB1 may lead to neuronal death and subsequently trigger inflammation (Homma et al., 2024).

In addition to inducing inflammation, HMGB1 also has a direct impact on the pathological process of PD. It can induce mitochondrial dysfunction and disrupts PTEN-induced kinase 1 (PINK1)/Parkin-mediated mitophagy. In addition, it can activate the HMGB1/TLR4/NF-κB pathway. For example, in the Zebrafish model induced by 1-methyl-4-phenyl-1,2,3,6-tetrahydropyridine (MPTP), HMGB1 acts as a key bridge between mitochondrial dysfunction and neuroinflammation (Razali et al., 2023). HMGB1 also plays a dual role in autophagy. It can promote autophagy initiation by binding to Beclin-1. However, when exposed to the toxin paraquat, the interaction between HMGB1 and α-syn is stronger. This interaction not only drives the accumulation of α-syn but also leads to the competitive disruption of the HMGB1 Beclin1 complex by the accumulated α-syn. This process impairs autophagic activity and further exacerbates the accumulation of α-syn (Wang et al., 2022). On the contrary, upregulating the expression of HMGB1 or Beclin-1 can promote the clearance of α-syn. Cory B has been shown to exert neuroprotective effects by enhancing the binding between HMGB1/2 and Beclin1, thereby activating autophagy and reducing α-syn load. This discovery supports HMGB1/2 as a potential target for PD therapy (Zhu et al., 2023b). Furthermore, the release of HMGB1 can trigger non-motor symptoms by activating spinal cord microglia (Sato et al., 2022).

Preclinical studies have explored multiple interventions targeting HMGB1 for PD (Paudel et al., 2020b; Mao et al., 2022). In the MPTP model of Zebrafish, glycyrrhizin inhibits HMGB1 and related inflammatory markers. This compound protects dopaminergic neurons and improves motor function by interacting with HMGB1, TLR4, and RAGE, thereby alleviating PD-like symptoms (Ren et al., 2022). Cilostazol exhibits neuroprotective effects in rotenone-induced PD rats, mediated by the inhibition of the HMGB1/TLR4 axis and the activation of the Nrf2/HO-1 and PI3K/Akt pathways. This process ultimately improves motor behavior, restores dopamine levels, and enhances tyrosine hydroxylase activity (El-Sayed et al., 2023). Oxymatrine alleviates motor deficits and neuroinflammation in MPTP-treated mice by targeting the HMGB1/TLR4/NF-κB pathway. Notably, overexpression of CathD can reverse these protective effects (Gan et al., 2020). Agmatine improves PD symptoms and mitigates levodopa-induced movement disorders by blocking the HMGB1/RAGE/TLR4/MyD88/NF-κB signaling cascade and activating Nrf2. It also reduces inflammation, alleviates oxidative stress, diminishes motor abnormalities, and protects dopaminergic neurons (Azar et al., 2022). Repetitive transcranial magnetic stimulation can downregulate the HMGB1/TLR4 signaling pathway in 6-hydroxydopamine-induced PD rats, which reduces neuroinflammation, enhances motor symmetry, and restores the expression of tyrosine hydroxylase (Han et al., 2023). In the rotenone mouse model, the combination of canagliflozin and levodopa/carbidopa inhibits RAGE/HMGB1/NF-κB activity, supporting its potential as an adjunctive therapy for PD (Elkady et al., 2024). A bibliometric analysis of 47 articles published between 2011 and 2022 showed that research on HMGB1 in PD is growing at a rate of 19.35% per year. This research primarily focuses on neuroinflammation, microglia, and RAGE. Since 2017, the relationship between HMGB1, PD, and microglial activation has gained prominence, providing direction for future research (Han et al., 2023). Overall, HMGB1 is increasingly recognized as a key mediator of PD, involved in critical processes such as neuroinflammatory responses, neurodegeneration, mitochondrial damage, and α-syn aggregation. These findings further emphasize its potential as a therapeutic target.

### Other neurodegenerative diseases

HMGB1 exerts pro-inflammatory DAMP effects through receptors such as RAGE and TLR4, triggering neuroinflammation. During the early stages of brain development, it supports neurite growth; however, in adult neurological diseases, it exacerbates pathological conditions. Studies have shown that the expression level of HMGB1 is increased in AD, PD, and MS, while it is decreased in HD and spinocerebellar ataxia. In tau proteinopathies, including AD and FTD, TauO trigger the release of HMGB1 from astrocytes and induce the senescence-associated secretory phenotype. This reaction exacerbates neuroinflammation and promotes paracrine senescence (Gomes et al., 2019; Gaikwad et al., 2021). The use of drugs such as pyruvate ethyl ester or glycyrrhizic acid to inhibit the release of HMGB1 can block key senescence-associated secretory phenotype pathways, including p38-MAPK and NF-κB, reduce the accumulation of tau protein oligomers and senescent cells, and improve cognitive ability in hTau mouse models (Gomes et al., 2019; Gaikwad et al., 2021). These findings collectively confirm that HMGB1-mediated senescence is an important common mechanism in tau proteinopathies (Jin et al., 2021).

ALS is a fatal motor neuron disease driven by neuroinflammation and oxidative stress, involving the HMGB1 signaling pathway through RAGE and TLR4. In the spinal cords of patients, the expression of HMGB1 and its receptors (RAGE and TLR4) is upregulated, and it has been found that ligands such as S100B and advanced glycation end products are also elevated (Juranek et al., 2015). In *SOD1*^G93A^ transgenic mice (a preclinical ALS model), HMGB1 translocates from motor neuron nuclei to the cytoplasm, suggesting extracellular release. However, astrocytes expressing mutant SOD1 fail to upregulate neurotrophic factors [brain-derived neurotrophic factor (BDNF), and glial cell-derived neurotrophic factor (GDNF)] in response to HMGB1, unlike healthy astrocytes, disrupting neuroprotective signaling (Wang et al., 2024a). In preclinical studies, targeting the HMGB1/RAGE/TLR4 pathway shows promise, as inhibiting these axes in the model can reduce motor neuron death and delay disease progression (Paudel et al., 2020a). However, treating SOD1^G93A^ mice with an anti-HMGB1 antibody (2G7) only briefly improves early motor functions, such as hindlimb grip strength, and moderately reduces the expression levels of pro-inflammatory markers, such as TNF-α and C5aR1, but does not prolong survival or alleviate glial activation, indicating its limited efficacy (Paudel et al., 2020a; Tian et al., 2023). Stage-specific analysis in SOD1ᴳ⁹³ᴬ mice further linked HMGB1 upregulation to symptom stages and revealed increased NF-κB and pro-inflammatory cytokines, supporting the role of HMGB1 as a target related to disease stages (Gomes et al., 2019).

HD is a monogenic neurodegenerative disorder driven by the expansion of CAG repeat sequences in the *HTT* gene, and HMGB1 contributes to its pathological processes (Angelopoulou et al., 2020). Nuclear HMGB1 aids in DNA repair and regulates CAG repeat instability (Angelopoulou et al., 2020). Under oxidative stress, cytoplasmic HMGB1 interacts with the mutant Huntington protein (mHTT) and promotes its translocation to the nucleus (Angelopoulou et al., 2020). Conversely, studies in SH-SY5Y cells and primary striatal neurons have shown that HMGB1 exerts neuroprotective effects: it inhibits mHTT aggregation—likely via autophagy regulation—and reduces polyglutamine (polyQ) toxicity by binding directly to expanded polyQ tracts (Angelopoulou et al., 2020). Extracellularly, HMGB1 activates RAGE/TLR2-4 signaling, triggering neuroinflammation and apoptosis in HD (Gendy et al., 2023). Notably, glycyrrhizin—a HMGB1 inhibitor—alleviates 3-nitropropionic acid (3-NP)-induced HD-like pathology in rats. It does so by suppressing the HMGB1/TLR4/NF-κB pathway, reducing oxidative stress, and boosting neurotrophic factors (BDNF and Bcl-2), underscoring HMGB1 as an actionable therapeutic target for HD (Gendy et al., 2023). In spinocerebellar ataxia type 1 (SCA1), mutant ataxin-1 impairs HMGB1 function. Viral or transgenic HMGB1 complementation ameliorates motor deficits and extends lifespan in SCA1 mice through novel mitochondrial DNA repair (Gehrking et al., 2011; Ito et al., 2015). In SCA17, caused by polyQ-expanded TATA-binding protein (TBP), HMGB1 is sequestered into mutant TBP aggregates. While the overexpression of HMGB1 mitigates TBP aggregation, regulates HSPA5 transcription, and enhances autophagy, the loss of HMGB1 function is associated with impaired neurite outgrowth (Lee et al., 2014a). In dementia with Lewy bodies, HMGB1 binds to aggregated α-syn and localizes to Lewy bodies. This sequestration is suggested to interfere with transcriptional regulation and contribute to neurodegenerative pathology (Lindersson et al., 2004).

**Figure 3:**
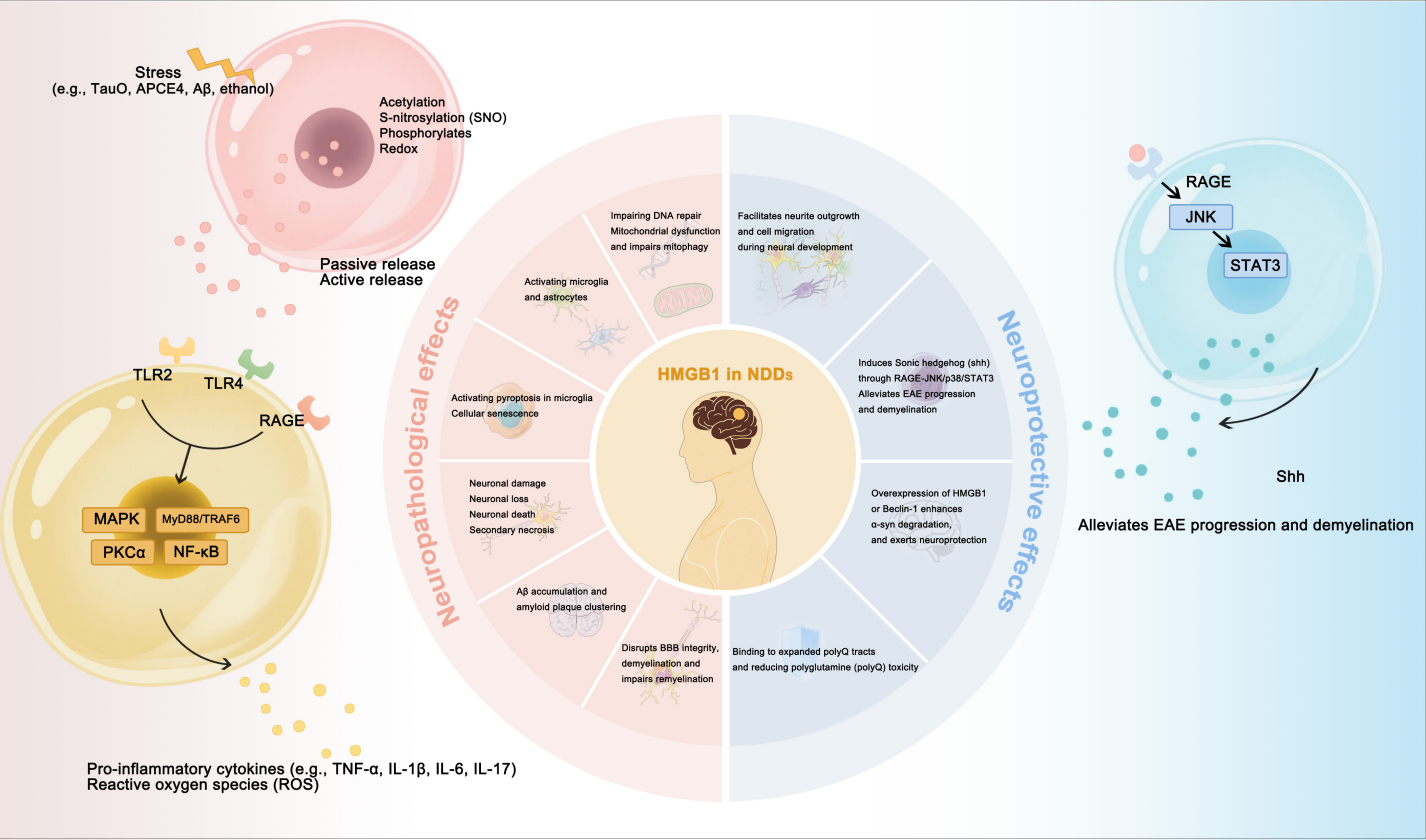
Mechanisms of the role of HMGB1 in NDDs. Following cellular stress, the alarmin HMGB1 undergoes PTMs—acetylation, S-nitrosylation, phosphorylation or redox shifts—that govern its passive or active release from neurons, astrocytes and microglia. Extracellular HMGB1 engages pattern-recognition receptors (TLR2, TLR4, RAGE) to provoke pro-inflammatory cytokine release (TNF-α, IL-1β, IL-6, IL-17) and reactive ROS. Pathologically, HMGB1 activation disrupts blood–brain-barrier integrity (via tight-junction loss), triggers microglial/astrocytic reactivity, induces pyroptosis, impairs remyelination, promotes Aβ aggregation and amyloid plaque formation, accelerates secondary necrosis, compromises DNA repair, drives cellular senescence, and precipitates mitochondrial dysfunction and mitophagy failure—culminating in neuronal loss and death. Conversely, context-dependent HMGB1 signaling can be neuroprotective: it induces Shh to attenuate EAE progression and demyelination, facilitates neurite outgrowth and neural migration, cooperates with Beclin-1 to enhance α-synuclein degradation, and mitigates polyglutamine toxicity through direct binding to expanded polyQ tracts. Aβ: Amyloid-beta; EAE: experimental autoimmune encephalomyelitis; HMGB1: high-mobility group box 1; IL-1β: interleukin-1β; IL-6: interleukin-6; IL-17: interleukin-17; MAPK: mitogen-activated protein kinase; NDDs: neurodegenerative diseases; NF-κB: nuclear factor kappa-light-chain-enhancer of activated B cells; PKCα: protein kinase C alpha; RAGE: receptor for advanced glycation end products; ROS: reactive oxygen species; STAT3: signal transducer and activator of transcription 3; TauO: Tau oligomers; TLR2: Toll-like receptor 2; TLR4: Toll-like receptor 4; TNF-α: tumor necrosis factor-alpha; TRAF6: TNF receptor-associated factor 6.

While HMGB1 and its receptors (RAGE, TLR4) are validated preclinical targets in NDDs, challenges remain. Clinical translation is hindered by transient efficacy observed in ALS models, a lack of validated HMGB1 biomarkers, and the context-dependent roles of HMGB1 (e.g., protective versus pathogenic in HD). Overall, HMGB1 has complex effects on the precise regulation of NDDs (**Figure 3**). Further studies are needed to clarify tissue-specific and stage-specific mechanisms, optimize HMGB1-targeted therapies (e.g., glycyrrhizin derivatives and anti-HMGB1 antibodies), and validate their safety and efficacy in human trials. The expression levels and mechanisms of HMGB1 in different NDDs are summarized in **Additional Table 2**.

**Additional Table 2:** Expression levels and mechanisms of action of HMGB1 in different NDDs

NDD	HMGB1 expression	Key mechanism	Reference
MS	Elevated in peripheral blood, CSF, serum; serum correlates with disease severity; peaks in EAE onset; nuclear-to-cytoplasmic translocation in CNS cells.	Amplifies neuroinflammation via microglia/astrocyte activation (TNF-α, IL-1β, IL-6). Disrupts BBB via OPC-derived HMGB1, enabling T-cell infiltration. Inhibits remyelination via TLR2/NF-κB.	Chu et al., 2021; Kim et al., 2025a, b Kim et al., 2025 a, b Rouillard et al., 2022
		Activates TLR4/NF-κB axis (MyD88/TRAF6 pathway).	Gendy et al., 2023
		Promotes pyroptosis in microglia and other cells via the TLR4/NF-κB pathway.	Feng et al., 2025
AD	Co-localizes with Aβ plaques; elevated in CSF, serum, and MCI/AD patients; nuclear-to-cytoplasmic shift in Tg-APP/PSl models (precedes Aβ deposition).	IL-33 deficiency enhances astrocyte-derived HMGB1 release (via acetylation); binds RAGE, induces shh via JNK/p38/STAT3. Links Aβ/Tau pathology to neuroinflammation via TLR4/RAGE/NF-κB. Induces TRIAD necrosis via PKCa/Ku70. TauO induces HMGB1 release, driving p38-MAPK/NF-κB-mediated senescence.	Xiao et al., 2021, 2025 Rouillard et al., 2022 Paudel et al., 2020 Tanaka et al., 2021 Gaikwad et al., 2021
		APOE4 accelerates HMGB1 release, exacerbating Tauopathy.	Koutsodendris et al., 2023
		Disrupts BBB (synergizes with thrombin).	Festoff et al., 2016; Nishibori et al., 2020
		Intracellular Aβ induces primary TRIAD necrosis; released HMGB 1 propagates secondary necrosis and plaque clustering. Linked to astrocytic senescence; regulated by environmental factors via neuroimmune signaling.	Okazawa et al., 2021; Homma et al., 2024 Fisher et al., 2025; Sha et al., 2025
PD	Elevated in serum (correlates with disease duration, UPDRS), CSF, and substantia nigra; Co-upregulated with TLR4 in advanced PD.	Drives neuroinflammation via TLR4/RAGE/NF-κB (pro-inflammatory cytokines, ROS). S-nitrosylation (Cys106) and SIRT2-mediated translocation promote release. Cytoplasmic translocation (regulated by SIRT2 deacetylation) promotes release and neuroinflammation.	Yang et al., 2018; Gan et al., 2020; Razali et al., 2023 Liang et al., 2024; Xing et al., 2024 Xing et al., 2024
		May contribute to neuronal death via TRIAD necrosis.	Homma et al., 2024
		Impairs mitophagy (PINK1/Parkin) and autophagy (α-syn clearance).	Wang et al., 2022
		Dual role: initiates via Beclin-1; dysregulation with a-syn disrupts complex (impairs flux); overexpression/Cory B enhances α-syn degradation. Contributes to non-motor symptoms (e.g., pain).	Wang et al., 2022; Zhu et al., 2023 Sato et al., 2022
Others	Tauopathies: Upregulated in senescent astrocytes.	Tauopathies: TauO-induced HMGB1 release activates SASP (p38-MAPK/NF-κB).	Gomes et al., 2019; Gaikwad et al., 2021
	ALS: Increased in spinal cords; nuclear-to-cytoplasmic shift in SOD1^G93A^ mice.	ALS: TLR4/RAGE signaling; mutant SOD1 astrocytes lose neurotrophic response.	Juranek et al., 2015; Brambilla et al., 2018; Paudel et al., 2020
	HD: Reduced expression; nuclear-cytoplasmic redistribution.	HD: Dual role-promotes mHTT nuclear translocation; inhibits aggregation.	Angelopoulou et al., 2020; Gendy et al., 2023

This table focuses on the expression characteristics of HMGB1 in four major categories of NDDs (MS, AD, PD, and other conditions such as Tauopathies, ALS, and HD) and its key mechanisms of action in disease progression. The research clarifies that HMGB1 exhibits different expression patterns (elevation, reduction, subcellular localization shift) in various NDDs and contributes to disease development by regulating neuroinflammation, disrupting the blood-brain barrier, inhibiting cell regeneration, inducing cell death, and impairing protein clearance. In terms of application significance, this provides important clues for the diagnosis and treatment of NDDs. For example, the expression level of HMGB1 in body fluids (serum, CSF) may serve as a potential diagnostic marker for these diseases, and targeting the HMGBl-related signaling pathways (e.g., TLR4/NF-κB, RAGE) could represent a new therapeutic direction for alleviating neurodegenerative lesions. AD: Alzheimer’s disease; amyloid-beta; BBB: blood-brain barrier; CNS: central nervous system; CSF: cerebrospinal fluid; EAE: experimental autoimmune encephalomyelitis; HD: Huntington’s disease; HMGB1: high mobility group box 1; IL-1β: interleukin 1 beta; IL-33: interleukin 33; IL-6: interleukin 6; MCI: mild cognitive impairment; mHTT: mutant huntingtin; NDD: neurodegenerative disease; NF-κB: nuclear factor kappa B; p38-MAPK: p38 mitogen-activated protein kinase; PINK1: PTEN induced kinase 1; RAGE: receptor for advanced glycation end products; ROS: reactive oxygen species; SASP: senescence-associated secretory phenotype; SODI: superoxide dismutase 1; SIRT2: Sirtuin 2; Tg-APP/PS1: transgenic amyloid precursor protein/presenilin 1; TLR2: Toll-like receptor 2; TLR4: Toll-like receptor 4; TNF-α: tumor necrosis factor alpha; UPDRS: unified Parkinson’s disease rating scale; α-syn: alpha-synuclein.

## Therapeutic Potential of Targeting High Mobility Group Box 1 and Its Post-translational Modifications in Neurodegenerative Diseases

### Strategies to inhibit high mobility group box 1 release

HMGB1 acts as a critical driver of neuroinflammation in neurodegenerative pathologies. Its release from neurons, microglia, and astrocytes amplifies detrimental inflammatory cascades primarily through RAGE signaling, contributing to BBB disruption and accelerated neuronal loss (You et al., 2023; Hisaoka-Nakashima et al., 2024). Consequently, inhibiting HMGB1 release or neutralizing its extracellular activity presents a promising therapeutic avenue. The key strategies include the following:

#### Neutralizing antibodies

Directly targeting extracellular HMGB1 with specific antibodies reduces pro-inflammatory microglial activation via the RAGE-NF-κB pathway, decreases neuronal loss and demyelination, improves functional recovery in models such spinal cord injury (SCI) (Fan et al., 2020), and protects BBB integrity by preventing HMGB1-induced phosphorylation of VE-cadherin in endothelial cells, which is relevant in viral neuroinflammation and AD models (Festoff et al., 2016). In HIV-associated neurocognitive disorders, HMGB1 in CSF serves as an early biomarker of neurocognitive impairment, and is correlated with immune activation and viral persistence (Gougeon, 2017). Critically, a novel anti-HMGB1 mAb administered subcutaneously suppressed neurite degeneration and reversed cognitive deficits in AD model mice via dual inhibition of Aβ polymerization and HMGB1-driven actin destabilization (Fujita et al., 2016). Anti-RAGE antibodies shift microglia toward an anti-inflammatory phenotype in SCI (Fan et al., 2020), but show limited efficacy in ALS; transient improvement in motor function without survival benefit in SOD1G93A mice suggests compensatory pathways or insufficient CNS penetration (Fan et al., 2020).

#### Small molecule inhibitors

The ethyl pyruvate and glycyrrhizic acid can block TauO-induced astrocytic HMGB1 release, suppressing p38/NF-κB-mediated senescence and cognitive decline in AD/FTD (Gaikwad et al., 2021). Pharmacological blockade of glucocorticoid receptors prevents stress-induced glucocorticoids from triggering HMGB1 nuclear-to-cytoplasmic translocation and release from microglia, thereby mitigating associated neuroinflammation linked to diseases such as depression and dementia (Hisaoka-Nakashima et al., 2024). δ-Opioid receptor agonists (e.g., UFP-512) inhibit HMGB1 secretion and NF-κB activation, reducing Aβ pathology and microglial hyperactivation in AD (Xu et al., 2025). Inhibitors of autophagy/lysosomal function (e.g., Bafilomycin A1) suppress the autophagy-dependent release of HMGB1 occurring during ferroptosis (Wen et al., 2019), a cell death process implicated in neurodegeneration or gasdermin D-mediated pyroptosis (Tan et al., 2020; Volchuk et al., 2020).

#### Targeting neuronal-specific release mechanisms

Interventions focused on neurons include inhibiting HMGB1 release triggered by stress, neuronal depolarization, or cortical spreading depolarization, which occurs through packaging into small extracellular vesicles. Blocking the release of these small extracellular vesicles or their uptake by astrocytes (which activates astrocytic NF-κB) could prevent the initiation of neuroinflammation (Kaya et al., 2023). Neutralizing neuronal HMGB1 also shows efficacy in ameliorating neuroinflammation in models of nerve injury and pain, suggesting applicability to neurodegenerative pain syndromes (Yang et al., 2021a). Impaired neurotrophic response in SOD1G93A astrocytes to HMGB1 may underlie failed neuroprotection (Brambilla et al., 2018), highlighting the need for combinatorial approaches. Omega-3 polyunsaturated fatty acids (ω-3 PUFAs) modulate HMGB1/S100β-mediated neuroinflammation in depression, while HMGB1 release during TRIAD links neurodegeneration to neuroinflammation (Homma et al., 2024).

#### Regulating upstream regulatory factors

HMGB1 A-box protein can competitively block RAGE binding and restore CD200-CD200R-mediated microglial silencing, ultimately reducing the loss of dopaminergic neurons in PD (Tian et al., 2020). HMGB1-specific inhibitors for APOE4-associated AD can prevent HMGB1 translocation in neurons and alleviate Tau pathology, gliosis, and neurodegeneration (Koutsodendris et al., 2023). It is noteworthy that IL-33 regulates HMGB1 release in an interactive manner. HMGB1 drives astrocytes to secrete IL-33, while IL-33 inhibits HMGB1 acetylation and release by disrupting the binding of P300/CBP-related factors to HMGB1 in astrocytes (Homma et al., 2024; Xiao et al., 2025). This inhibitory axis is involved in the self-limiting phase of EAE (Homma et al., 2024) and is dysregulated in HIV-induced neuropathogenesis (Porto Silva et al., 2025), making IL-33 signaling a potential therapeutic target. Additionally, drugs that inhibit pyroptosis, such as Gasdermin D inhibitors like disulfiram, can prevent cell lysis and the subsequent passive release of HMGB1 (Tan et al., 2020; Volchuk et al., 2020). Collectively, these strategies targeting HMGB1 hold great potential for alleviating neuroinflammation and neuronal damage in NDD.

### Modulating high mobility group box 1 post-translational modifications for therapeutic benefits

HMGB1 is a critical driver of neuroinflammation and pathogenesis in NDDs. Its biological activity, including subcellular localization, secretion mechanisms, and extracellular inflammatory functions, is exquisitely regulated by specific PTMs, making it a promising therapeutic target (Tang et al., 2016).

Hyperacetylation within the nuclear localization sequences of HMGB1 drives its translocation from the nucleus to the cytoplasm, which is a prerequisite for its active release from immune cells such as macrophages and microglia (Tang et al., 2016; Richard et al., 2017). Once it enters the extracellular space, acetylated HMGB1 acts as a potent proinflammatory cytokine, orchestrating the innate and adaptive immune responses involved in neuroinflammation (Tang et al., 2016; Richard et al., 2017). The redox status of the cysteines (C23, C45, and C106) of HMGB1 dictates its extracellular activity. The formation of an intramolecular disulfide bond (C23–C45), facilitated in the nucleus by Peroxiredoxins I/II (PrxI/II), is crucial for its cytokine-inducing activity (Tang et al., 2016; Kwak et al., 2020). This disulfide HMGB1 is actively exported via CRM1 and secreted, a process essential for its proinflammatory signaling in inflammatory neurodegeneration (Tang et al., 2016; Kwak et al., 2020). Elevated lactate levels, which are common in metabolic stress and neuroinflammation, drive HMGB1 lactylation on lysine residues (Yang et al., 2022a). Lactylated HMGB1 promotes its exosomal release, a mechanism implicated in encephalopathies (Yang et al., 2022a). Enhancing regulators such as HSPA12A, which suppress glycolysis and downstream HMGB1 lactylation, could counter metabolic stress-induced HMGB1 release during neurodegeneration (Du et al., 2023).

In PD, iNOS-derived nitric oxide induces SNO at Cys106 of HMGB1 (SNO-HMGB1), which is essential and sufficient for inflammation-triggered HMGB1 secretion (Yang et al., 2022b). SNO-HMGB1 significantly enhances proinflammatory and neurodegenerative effects; its intranigral injection in mice induces chronic microglial activation, dopaminergic neurodegeneration, and PD-like locomotor deficits, dependent on Mac1 receptor interaction (Yang et al., 2022b). Inhibition of iNOS or mutation of Cys106 prevents HMGB1 release and neurotoxicity, highlighting SNO as a critical therapeutic target (Yang et al., 2022b). Cytoplasmic HMGB1 can be targeted for proteasomal degradation via ubiquitination. The E3 ubiquitin ligase RNF186 promotes K48- and K63-linked ubiquitination of cytoplasmic HMGB1, leading to its degradation (Du et al., 2024). Depletion of RNF186 increases cytoplasmic HMGB1 levels (Du et al., 2024). Conversely, the E3 ligase CHIP promotes HMGB1 ubiquitination and degradation, suppressing its pathological functions (Sun et al., 2022).

Therapeutic strategies targeting these PTMs include: blocking SNO (e.g., via iNOS inhibition or NO scavengers) to prevent HMGB1 release and neuroinflammation in PD (Yang et al., 2022b); modulating lactate pathways (e.g., targeting monocarboxylate transporters or the lactate receptor GPR81) or enhancing HSPA12A to reduce lactate-driven HMGB1 lactylation/acetylation and exosomal release (Yang et al., 2022a); enhancing ubiquitin-mediated degradation pathways by modulating E3 ligases such as CHIP or inhibiting deubiquitinating enzymes to reduce cytoplasmic HMGB1 availability (Sun et al., 2022; Du et al., 2024), and using small molecules such as inflachromene that bind HMGB1 and block its cytoplasmic translocation and release by perturbing PTMs (Lee et al., 2014b). Neutralizing extracellular HMGB1 or blocking its receptors (e.g., RAGE, TLR4, and Mac1) remains viable; blocking the HMGB1-Mac1 interaction is particularly relevant for PD (Yang et al., 2022b; Meng et al., 2024).

**Figure 4:**
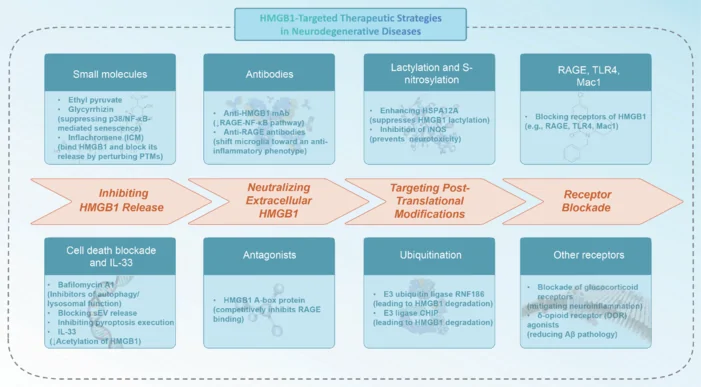
HMGB1-targeted therapeutic strategies in NDDs. Graphical summary of therapeutic strategies targeting HMGB1 in NDDs. Four major approaches are illustrated: inhibiting HMGB1 release (via small molecules, blockade of cell death, and modulation of IL-33), neutralizing extracellular HMGB1 (using antibodies and antagonists), targeting post-translational modifications (including lactylation, S-nitrosylation, and ubiquitination), and receptor blockade (focusing on RAGE, TLR4, Mac1, and other receptors). Each strategy encompasses specific molecular interventions aimed at modulating HMGB1-related pathological pathways for potential treatment of NDDs. HMGB1: High-mobility group box 1; IL-33: interleukin-33; iNOS: inducible nitric oxide synthase; Mac1: macrophage-1 antigen; NDDs: neurodegenerative diseases; RAGE: receptor for advanced glycation end products; TLR4: Toll-like receptor 4.

In conclusion, the diverse PTMs of HMGB1 (acetylation, redox modifications, SNO, lactylation, and ubiquitination) act as molecular switches controlling its neuroinflammatory potential. Developing therapeutics that selectively modulate these PTMs–inhibiting pathogenic modifications (SNO, lactylation), promoting degradation (ubiquitination), or blocking release- offers novel avenues for mitigating neuroinflammation in NDDs. Future research must elucidate brain-specific regulatory pathways for HMGB1 PTMs and validate these targeted approaches in relevant disease models (**Figure 4**).

### Challenges and future directions in high mobility group box 1–based therapies

HMGB1 poses unique challenges for treating NDDs owing to its structural flexibility, functional diversity, and complex dual roles in physiological versus pathological processes. While HMGB1 is an attractive target, inhibiting it may disrupt essential physiological functions, such as infection control, dead cell clearance, and tissue repair (Kim et al., 2025b). Therefore, HMGB1 blockade must be highly specific and timed appropriately to avoid adverse impacts on innate immunity and healing mechanisms (Lv et al., 2024; Yan et al., 2024). A core challenge lies in distinguishing harmful, actively released HMGB1 from beneficial, passively released HMGB1 in the context of NDDs (Tian et al., 2023). Conflicting evidence underscores the complexity of HMGB1’s role in the brain. In the MPTP model, astrocyte-derived HMGB1 exerts neuroprotective effects by activating the c-JNK/RAGE signaling pathway, promoting tyrosine hydroxylase transcription, and reducing tyrosine hydroxylase loss (Ngadimon et al., 2024). However, this finding contradicts other studies that link HMGB1 translocation to neuronal injury. Furthermore, the intracellular functions of HMGB1—regulating transcription, autophagy, and apoptosis—suggest that long-term inhibition could carry unforeseen risks (Freitas-Andrade et al., 2023). The interaction between HMGB1 and α-syn disrupts autophagy, leading to toxic protein buildup and neuroinflammation; this implies that maintaining HMGB1 homeostasis may be preferable to broad receptor inhibition (Zhu et al., 2023b).

Another core impediment is the absence of a comprehensive understanding of the specific dynamic changes of HMGB1 under various disease conditions (Mao et al., 2022; Ngadimon et al., 2024). Whether HMGB1 is a major cause or an effect of neuronal damage has yet to be determined, and its variation according to disease course, cell type, and surrounding tissue remains unknown. These factors complicate the development of effective treatments. The advent of single-cell sequencing may provide an impetus for breakthroughs in studying AD and PD, allowing researchers to trace both convergent and diverging molecular pathways (Chen et al., 2025).

Existing HMGB1 inhibitors face further challenges in transplantation. Although monoclonal antibodies, glycyrrhetinic acid, and ethyl pyruvate demonstrate neuroprotective abilities in rodent models, their efficacy in humans is uncertain due to the subtle early manifestations of NDDs, unclear underlying mechanisms, and difficulties in drug delivery (Chen et al., 2021). Additionally, antibody-based therapeutics carry risks of hematological and autoimmune side effects, while glycyrrhetinic acid non-specifically blocks all receptor interactions (Wang et al., 2020). Furthermore, ethyl pyruvate only inhibits the release of HMGB1 from live cells (Gougeon et al., 2017). Regarding pathway-specific drugs, such as the TLRI/MD-2 inhibitor P5779, their feasibility is evident, but they do not account for the multifunctionality of HMGB1 (Sun et al., 2018). Notably, P5779, which suppresses the inflammatory response through DAMPs while sparing the innate immunity elicited by PAMPs, could be a promising therapeutic approach (Yang et al., 2015).

Further exploration of the active and passive release mechanisms of HMGB1 may lead to more precise therapeutic strategies. Investigating the intracellular transport of HMGB1, such as how mitochondrial peroxide levels affect its secretion, may help identify new drug targets. Studying the interactions between HMGB1 and TLRs, inflammasomes, pyroptosis, and ferroptosis could also reveal pathway-specific targets. As is well known, PTMs can regulate the transport, secretion, and dual functions of HMGB1 (Taira et al., 2013). Future research should focus on PTM regulatory pathways that shape the functional role of HMGB1. Structural studies (Cai et al., 2018; Tang et al., 2023) have identified ligand-binding sites on HMGB1 and validated its potential as a drug target (Yang et al., 2015). However, more work is needed to improve inhibitor design, particularly regarding redox-specific interactions (Tang et al., 2016; Yang et al., 2021b). The activity of HMGB1 is strictly controlled by the redox state of its cysteine residues, but existing inhibitors lack the specificity required for precise redox regulation. It is crucial to develop reagents that selectively target the pro-inflammatory disulfide form of HMGB1. This can be achieved by creating mutants or redox exchange inhibitors to minimize off-target effects. **Additional Table 3** shows more detailed information on the progress and challenges of HMGB1-related treatment methods.

**Additional Table 3:** Research progress and challenges of HMGB1-related therapeutic approaches

Therapeutic approaches	Research progress	Challenge	Reference
Inhibiting HMGB1 release	Neutralizing antibodies: Reduce pro-inflammatory microglial activation via the RAGE-NF-κB pathway, improve functional recovery, and shift microglia toward anti-inflammatory phenotype in SCI.	Antibodies carry hematological/autoimmune risks; anti-RAGE antibodies have limited efficacy in ALS.	Fan et al., 2020; Chen et al., 2021
	Small molecule inhibitors: Ethyl pyruvate and glycyrrhizic acid block astrocytic HMGB1 release; autophagy/lysosomal inhibitors suppress HMGB1 release in ferroptosis or pyroptosis.	Glycyrrhizin non-specifically blocks all receptor interactions; ethyl pyruvate only inhibits release from viable cells.	Gougeon et al., 2017; Tan et al., 2020; Volchuk et al., 2020; Wang et al., 2020; Gaikwad et al., 2021
	Targeting neuronal-specific release: Blocking sEV release or uptake halts neuroinflammation initiation.	Poor understanding of active versus passive HMGB1 release mechanisms and cell-type specificity of HMGB1 functions complicates targeting.	Volchuk et al., 2020; Kaya et al., 2023; Kwak et al., 2023
	Modulating upstream regulators: Pyroptosis inhibitors prevent passive HMGB1 release.	Unclear consequences of manipulating HMGB1 translocation versus secretion.	Tan et al., 2020; Volchuk et al., 2020
Modulating HMGB1 PTMs	Acetylation: Hyperacetylation drives nuclear-to-cytoplasmic translocation.	Limited understanding of brain-specific PTM regulatory pathways.	Tang et al., 2016; Richard et al., 2017
	Redox modifications: Disulfide HMGB1 (C23-C45) is proinflammatory.	Redox heterogeneity complicates targeting; current inhibitors lack redox selectivity.	Tang et al., 2016; Kwak et al., 2020
	Lactylation: Lactylated HMGB1 promotes exosomal release; modulating lactate pathways or HSPA12A reduces release.	PTM landscape in neurodegenerative brain regions is uncharted.	Taira et al., 2013; Du et al., 2023
	S-nitrosylation: Inhibiting iNOS or Cys106 mutation prevents HMGB1 release and neurotoxicity in PD Ubiquitination: E3 ligases (CHIP, RNF186) promote HMGB1 degradation.	Unclear interactions with lysosomal/mitochondrial dynamics.	Kwak et al., 2020; Yang et al., 2022a, b Sun et al., 2022; Du et al., 2024
	Small molecules (e.g., ICM) block translocation/release by perturbing PTMs.		Lee et al., 2014
New technologies for HMGB1 research	PTM-specific mass spectrometry: Enables quantification of site-specific HMGB1 PTMs; identifies PTM crosstalk.	Requires large sample volumes; high cost; needs specialized bioinformatics pipelines.	Wei et al., 2023; Ge et al., 2024
	scRNA-seq with HMGB1 co-detection: Profiles HMGB1 expression/signaling in individual CNS cells; uncovers cell-type-specific HMGB1 release in MS/AD.	Cannot directly detect HMGB1 PTMs; high technical variability; limited to fresh/frozen tissues.	Chuetal., 2021; Shaetal., 2025
	CRISPR-Cas9-based PTM site mutagenesis: Validates functional impact of specific PTM sites via point mutations; generates PTM-specific knockout models.	Off-target editing risks; cannot mimic dynamic PTM.	Liang et al., 2024; Xing et al., 2024
	*In vivo* HMGB1 biosensors: Monitors real-time HMGB1 redox state and subcellular localization in live EAE/PD models; evaluates inhibitor efficacy dynamically.	Limited to fluorescent readouts; biosensor expression may alter HMGB1 function.	Kwak et al., 2021; Wang et al., 2024
	Patient-derived iPSC organoids: Recapitulates NDD pathology to study the role of HMGB1 in early MCI/PD; tests PTM-specific drugs in human-relevant models.	Long culture time; limited vascularization.	Gaikwad et al., 2021; Fisher et al., 2025
General challenges for HMGBl-based therapies	HMGB1 is validated as a druggable target (e.g., aspirin inhibits HMGB1).	Dual roles of HMGB1: Inhibiting it may compromise regenerative inflammation, dead cell clearance, and tissue repair.	Kim et al., 2025a, b
	Preclinical evidence links HMGB 1 to neuroinflammation and neurodegeneration.	Unclear spatiotemporal dynamics and context-dependent roles; uncertainty whether HMGB1 is a primary driver or downstream consequence.	Fan et al., 2020; Yang et al., 2021a, b; Kaya et al., 2023; Ngadimonet al., 2024
		Intracellular HMGB1 regulates transcription, autophagy, and apoptosis; long-term inhibition risks are unknown.	Freitas-Andrade et al., 2023; Xue et al., 2023; Chen et al., 2024
		Existing inhibitors face translational barriers (subtle early pathology, agent-specific limitations).	Liu et al., 2023

This table summarizes the research progress of two major HMGBl-related therapeutic strategies, five core new technologies for HMGB1 research, and the general challenges faced by HMGBl-based therapies. The research progress indicates that neutralizing antibodies, small molecule inhibitors, and targeting upstream regulatory pathways can effectively inhibit HMGB1 release, while regulating specific PTMs can interfere with HMGB1 function. The integrated new technologies for HMGB1 research provide critical tools for addressing existing research gaps. Notably, the table emphasizes the need to integrate these new technologies with clinical research pipelines, highlighting the translational gap between technical advances in preclinical studies and practical applications in clinical settings. Additionally, it is confirmed that HMGB1 has druggable potential, and preclinical studies have verified its correlation with neuroinflammation and neurodegeneration. In terms ofapplication significance, this overview provides a comprehensive foundation for the development of HMGB1-targeted drugs, helping researchers clarify the direction of subsequent research (e.g., improving the specificity of small molecule inhibitors and exploring brain-specific PTM regulatory pathways) and rationally assess the potential risks of therapeutic strategies (e.g., avoiding adverse effects caused by the dual role of HMGB1). The general challenges identified also provide a basis for addressing the translational bottlenecks of HMGB1-based therapies. AD: Alzheimer's disease; ALS: amyotrophic lateral sclerosis; CHIP: carboxyl terminus of Hsc70-interacting protein; CNS: central nervous system; EAE: experimental autoimmune encephalomyelitis; HMGB1: high mobility group box 1; ICM: intracellular channel modulator; iNOS: inducible nitric oxide synthase; MS: multiple sclerosis; MCI: mild cognitive impairment; NDD: neurodegenerative diseases; PD: Parkinson’s disease; PTM: post-translational modification; RAGE: receptor for advanced glycation end products; SCI: spinal cord injury; sEV: small extracellular vesicle.

## Current Status of Clinical Translation and Application

HMGB1 has gradually become a marker for certain diseases and a target for treatment. In oncology, the University Hospital of Clermont-Ferrand (NCT06649474) is evaluating HMGB1 as a predictor of hepatotoxicity and neurotoxicity associated with oxaliplatin. In sepsis, elevated levels of HMGB1 in the late stage of the disease are associated with 28-day mortality, particularly in patients with diabetes or chronic obstructive pulmonary disease. Sivelestat, a neutrophil elastase inhibitor, reduces HMGB1 levels and improves cardiac function in patients with sepsis-induced acute respiratory distress syndrome and septic cardiomyopathy (Karakike et al., 2019). The University Hospital of Bordeaux (NCT04080453) is investigating the interaction between HMGB1 and platelets in septic shock. This study aims to elucidate the interaction between HMGB1 and coagulation during sepsis.

The combination of aromatherapy and music therapy can reduce HMGB1 and IL-6 levels, as well as alleviate pain and anxiety in patients with breast cancer (Deng et al., 2022). Additionally, the anterior quadratus lumborum block can reduce postoperative levels of HMGB1 and IL-6, which may help alleviate cognitive dysfunction in elderly patients after hip surgery (Zhu et al., 2023a). In organ transplantation, mild hypothermia reduces HMGB1 levels in brain-dead renal donors, thereby decreasing the risk of delayed graft function in recipients (Shan et al., 2020). Beyond these areas, multiple studies have also focused on HMGB1 under other conditions:

(1) NCT06971770 compares salivary HMGB1 levels between smokers and non-smokers with periodontal disease to validate its diagnostic utility.

(2) NCT03741738 investigates associations between HMGB1 and vitiligo activity.

(3) NCT01705353 examines the relationship between HMGB1 and post-stroke functional recovery.

(4) NCT02206412 evaluates HMGB1 as a prognostic biomarker in cardiac surgery.

Additionally, rectal indomethacin downregulates HMGB1 to prevent post-endoscopic retrograde cholangiopancreatography pancreatitis. Continuous renal replacement therap also reduces HMGB1 levels in severe acute pancreatitis, thereby boosting treatment efficacy (Gao et al., 2018). Most neurological studies on HMGB1 have focused on acute conditions or epilepsy, rather than chronic NDDs. In acute spinal cord injury, hyperbaric oxygen therapy downregulates HMGB1 and NF-κB, which reduces F-wave dispersion and improves motor function, as measured by ASIA/Frankel scores (Sun et al., 2019). For epilepsy, Assiut University (NCT05555537) is evaluating HMGB1 and miRNA223 as biomarkers for drug-resistant epilepsy. Preclinically, HMGB1 has been linked to ictogenesis through neuroinflammation (Auvin et al., 2017). In traumatic brain injury, drive pressure–guided individualized positive end-expiratory pressure, a lung-protective ventilation strategy, lowers serum HMGB1 and neuron-specific enolase, promoting neurological recovery without increasing intracranial pressure (Chen et al., 2024). Transauricular vagus nerve stimulation reduces HMGB1 and S100β levels in elderly patients after total joint arthroplasty, decreasing the incidence of delayed neurocognitive recovery (Zhu et al., 2024).

Preclinical studies connect HMGB1 to the pathogenesis of NDDs; however, direct clinical research on HMGB1 in NDDs remains limited. Current work on HMGB1 in NDDs is restricted to indirect correlations. For instance, in glioma, adenoviral delivery of Flt3L recruits dendritic cells, while HMGB1 boosts anti-tumor immunity (NCT01811992). Yet, this work centers on malignancy rather than degenerative processes (Lowenstein and Castro, 2018). HMGB1 faces major hurdles in the context of NDDs. First, no clinical trials have specifically assessed HMGB1 as a biomarker or therapeutic target in AD, PD, or MS. Second, existing HMGB1 inhibitors, such as glycyrrhizic acid and ethyl pyruvate, show poor BBB penetration, even though they have been investigated in non-neurological fields. Third, HMGB1 has context-dependent functions: it can act as both a pro-inflammatory mediator and a neuroprotective factor during chronic neurodegeneration, complicating therapeutic targeting. In summary, although HMGB1 remains clinically unvalidated in NDDs, targeted research is critical to harnessing it as both a biomarker and a therapeutic strategy.

## Discussion

This review examines how the dual functions of HMGB1 in NDDs are precisely regulated through its PTMs. Unlike previous comprehensive reviews on HMGB1 in CNS diseases, this work highlights PTMs as pivotal regulators, investigating their impact on the roles of HMGB1 in both neuroinflammation and neuroprotection. These insights provide a fresh perspective for developing targeted therapies focused on neural regeneration and repair. Additionally, this review synthesizes the molecular mechanisms that underpin the dual role of HMGB1 as a nuclear stabilizer and an inflammatory signal. PTMs, including acetylation, SNO, and disulfide bond formation, actively promote the pathological release of HMGB1 and subsequent receptor activation in NDDs. In contrast, the role of HMGB1 in sustaining autophagy and DNA repair reflects its protective function. A key translational insight from this work is that targeting HMGB1 through its PTMs could lead to the development of therapeutic strategies for NDDs. Unlike broad HMGB1 inhibition, which may disrupt neuroprotective functions, PTM-specific interventions allow for the precise suppression of harmful HMGB1 isoforms, such as S-nitrosylated forms in PD or disulfide-HMGB1 in MS, while sparing their reparative roles in autophagy and DNA repair. PTM-based HMGB1 biomarkers may facilitate early diagnosis and patient stratification. Meanwhile, brain-penetrant inhibitors targeting specific modifying enzymes (e.g., iNOS), along with isoform-selective antibodies, offer promising routes to disrupt neuroinflammatory cycles and foster a pro-regenerative environment.

While current research has begun to uncover the pathways regulating HMGB1 PTMs, key challenges persist. Past work has focused on deciphering the redox code of HMGB1 and its acetylation-driven secretion mechanisms. However, the lack of clinical-grade tools to image or quantify these PTM isoforms in patients remains a critical barrier. Furthermore, the potential synergistic or antagonistic effects of multiple PTMs acting on a single HMGB1 molecule simultaneously have not been studied. Another significant challenge is achieving sustained, cell-specific drug delivery across the BBB. For instance, precise targeting of SNO modifications in microglia while avoiding interference with HMGB1 in neurons is essential.

Future research may focus on several key aspects. It is urgent to develop highly specific monoclonal antibodies or nanobodies that can distinguish HMGB1 PTM subtypes, such as reduced forms, disulfides, and SNO. Additionally, small molecules need to be designed to cross the BBB and inhibit specific PTM enzymes, or to covalently bind and inactivate key cysteine residues (such as Cys106). Determining the optimal treatment window for intervention is crucial for maximizing regenerative effects. Finally, investigating combination therapies that simultaneously target pathogenic HMGB1 PTMs and enhance protective pathways, such as promoting autophagy by reducing HMGB1 levels, can provide a multifunctional strategy to prevent neurodegeneration and support repair.

## Limitations

Although this review provides a systematic summary of the existing findings on PTMs of HMGB1 in NDDs, there are still several limitations. Research in this field primarily relies on preclinical models such as EAE, MPTP-induced models, and APP/PS1 transgenic mice. While these models have research value, they have not been able to fully replicate the complex and chronic progression of human NDDs. Additionally, there is limited data on the localization and quantification of HMGB1 PTMs in the brains of human patients at different disease stages, which poses a challenge for translational research. Furthermore, most of the functional results related to HMGB1 PTMs are mainly inferred from *in vitro* studies or from broad regulations of HMGB1 activity. Efforts to validate these inferred functions have been hindered by the lack of tools, such as antibodies that can specifically detect particular PTM subtypes *in vivo* and in clinical samples.

Moreover, extensive cross-regulation exists between distinct PTMs, and these modifications exert cell-type-specific effects. This not only complicates mechanistic investigations but also suggests that our current models may oversimplify the actual biological context.

## Conclusion

This review summarizes the key roles of HMGB1 and its PTMs in the pathogenesis of NDDs. Targeting HMGB1 through its PTMs has the potential to reshape strategies for neural regeneration. Further research on the molecular mechanisms of the PTM-mediated regulation of HMGB1 will advance the design of targeted therapies. In the future, treatments will not only alleviate neuroinflammation but also promote nerve regeneration, providing new hope for reversing the progression of NDDs.

## Additional files:

***Additional Table 1:*** *Types of PTMs of HMGB1 and their functional impacts.*

***Additional Table 2:*** *Expression levels and mechanisms of action of HMGB1 in different NDDs.*

***Additional Table 3:*** *Research progress and challenges of HMGB1-related therapeutic approaches.*

## Data Availability

*All relevant data are within the paper and its Additional files.*
